# Less cooperative belowground plant mutualists negatively affect aboveground herbivore growth and survival

**DOI:** 10.1007/s00442-026-05966-7

**Published:** 2026-09-04

**Authors:** Annaliese H. Wargin, Katy D. Heath, Jennifer A. Lau, Adam G. Dolezal, Alexandra N. Harmon-Threatt

**Affiliations:** 1 Department of Entomology, University of Illinois Urbana-Champaign, Urbana , 61820 IL USA; 2 Department of Plant Biology, University of Illinois Urbana-Champaign, Urbana , 61820 IL USA; 3 Department of Biology, Indiana University, Indiana, 47405 IN USA

**Keywords:** Mutualism, Herbivory, Plant defense, Nitrogen, Symbiosis

## Abstract

Resource mutualists like nitrogen (N)-fixing rhizobia can improve the quality of plants available to higher trophic levels, particularly primary consumers like insect herbivores. However, there is substantial variation in mutualist quality. How this variation in mutualist quality affects higher trophic levels, such as herbivores, is unclear, as high-quality resource mutualists could increase both leaf nutrient content, which should improve herbivore growth, but also plant chemical defenses, which are expected to negatively impact herbivores. We evaluated the effect of rhizobium strains of varying mutualist quality on the survival and development of a generalist insect herbivore, the beet armyworm (*Spodoptera exigua*), reared on alsike clover (*Trifolium hybridum*). We inoculated *T. hybridum* with cooperative (“high-quality”) and less cooperative (“low-quality”) rhizobia strains, measured plant growth, nutrients, and metabolites, and fed the leaves to *S. exigua*. We found that high-quality rhizobium strains significantly improved *S. exigua* survival, but also increased development time compared to low-quality strains. Furthermore, some developmental traits, such as pupal weight and adult wing area, increased in high-quality treatments. Inoculation with strains of varying quality impacted *T. hybridum* nutrition and phytochemistry, suggesting that both could mediate herbivore responses to mutualist quality. The effects of rhizobium genetic variation on herbivores exceeded the effects of N-fertilization alone: *S. exigua* fed leaves from uninoculated, fertilized plants had decreased survival compared to caterpillars fed leaves from high-quality partners, implying that N deposition into ecosystems may not fully replace benefits provided by rhizobia to higher trophic levels.

**Supplementary Information:**

The online version contains supplementary material available at https://doi.org/10.1007/s00442-026-05966-7.

## Introduction

Interactions between plants and soil microbes are critical to the structuring of plant and animal communities in natural ecosystems (Reynolds et al. 2003; Hines et al. 2006; Heinen et al. 2018; Yan et al. 2022). Microbial mutualists which help facilitate resource acquisition, in particular, can have large effects on plant communities that potentially scale to affect higher trophic levels by either changing plant community composition (Keller 2014; Frouz et al. 2016; Zhou et al. 2022) or by modifying host plant quality immediately available to insect herbivores (Bezemer et al. 2005; Koricheva et al. 2009; Bustos-Segura et al. 2024). Yet, there is substantial variation in mutualist quality (Heath and Stinchcombe 2014; Weese et al. 2015). As a result, similar to how genetic variation in plants propagates through food webs to influence higher trophic levels (Crutsinger 2016), genetic variation in mutualists can affect plant traits, potentially in ways that scale up to affect herbivore growth and fitness.

Rhizobium bacteria, which provide legume plants with biologically available nitrogen (N) in exchange for plant photosynthetic products, can influence host plant biomass, nutrition, and defense (Lal and Khanna 1996; Rashid and Chung 2017; Rasmann et al. 2017; Allito et al. 2021; Basu et al. 2022). As insect herbivores are highly dependent on N for growth (Mattson 1980; Scriber and Slansky 1981), the simple expectation is that rhizobia would increase both plant quantity and quality for herbivores; yet, herbivore responses to rhizobia are variable. Some research has found that herbivores prefer to feed on rhizobium-inoculated plants, and show improved growth, abundance, and performance when fed tissues from these plants (Kempel et al. 2009; Katayama et al. 2011a, b; Dean et al. 2014; Keller et al. 2018; Godschalx et al. 2023; Bustos-Segura et al. 2024). These preferences and positive responses are usually correlated with elevated N content in rhizobium-inoculated plants (Pitcairn et al. 1998; Throop and Lerdau 2004). However, some studies find the opposite effect, with herbivores preferring uninoculated plants (Wilson and Stinner 1984; Thamer et al. 2011; Basu et al. 2022), or showing no response to plants inoculated with rhizobia (Heath and Lau 2011). Negative herbivore responses are often attributed to improved plant defense as, in many legume species, association with rhizobia results in an upregulation of nitrogen-based secondary defense compounds and repellant volatile cues (Edwards and Singh 2006; Ballhorn et al. 2013; Dean et al. 2014).

Variable herbivore responses to the presence or absence of rhizobia reveal a complex interaction between mutualist, plant, and herbivore, with some variation potentially resulting from variation in rhizobium mutualist quality. Rhizobium strains exist on a continuum from beneficial to uncooperative or even exploitative, in terms of their effects on plant growth, fitness, or N-fixation (Parker 1995; McInnes et al. 2004; Sachs and Simms 2008; Bromfield et al. 2010; Heath 2010; Weese et al. 2015; Gano-Cohen et al. 2019; Bamba et al. 2020; Zaw et al. 2021). This variation is likely to affect interactions with herbivores in ways similar to the presence or absence of mutualists. Herbivory is often studied in the context of the legume-rhizobium mutualism as a component of mutualism theory, given that herbivores act as a third party which applies selective pressure on the plant host and affects the costs and benefits of resource exchange (Heath and Lau 2011; Simonsen and Stinchcombe 2014; Thompson and Lamp 2021; Cassidy et al. 2022). Studies specifically investigating how herbivores themselves, rather than legumes, respond to variation in mutualist quality are sparse, but reveal a complex relationship between the three organisms. For example, past research has found that rhizobium strain identity can affect herbivore preference for a specific host plant (Dean et al. 2009; Simonsen and Stinchcombe 2014), with more beneficial strains increasing herbivore abundance or damage. How mutualist variability impacts herbivores beyond preference for or damage on a host plant, however, has rarely been explored. Given that insect herbivores are not simply intermediaries in the legume-rhizobium mutualism, but also critical to the structuring and functioning of food webs and greater ecosystem processes (Huntly 1991; Chapman et al. 2003; Gandhi and Herms 2010; Yang and Gratton 2014; Agrawal and Maron 2022), further investigation into how herbivores respond developmentally to variation in host plant rhizobia is necessary to disentangle the effects of plant-microbe mutualisms on higher trophic levels.

In this study, we determine the effects of rhizobium mutualist genetic variation on the survival and development of a generalist insect herbivore, the beet armyworm (*Spodoptera exigua*), as well as host plant traits relevant to herbivores (e.g., leaf N content and plant phytochemistry). By measuring rhizobium effects on both plant tissue nutrient content and plant metabolites, we aim to investigate these potentially contrasting effects on herbivores. We also compare these effects to an uninoculated, fertilized treatment to determine whether rhizobia have impacts above and beyond plant N availability.

## Materials and methods

### Study system

We used six strains of *Rhizobium leguminosarum* bacteria to inoculate *Trifolium hybridum* in a greenhouse experiment. All strains were a subset of those previously isolated from a nitrogen addition study at the Kellogg Biological Station Long Term Ecological Research site in Michigan, USA (Weese et al. 2015). Weese et al. (2015) isolated *R. leguminosarum* from nitrogen addition plots and control plots and ranked strains in terms of partner quality based on their growth effects on three species of clover (*Trifolium repens*, *Trifolium pratense*, and *Trifolium hybridum*). From this strain library, we selected three “high-quality” strains from the same identified genospecies (Vereau Gorbitz et al. 2025) that were ranked as the most cooperative or beneficial partners (strains HQ56, HQ108, HQ461), and three “low-quality” strains that were ranked as the least cooperative partners (strains LQ262, LQ651, LQ699) (Supplemental Table 1). Strains were named based on their numerical designations by Weese et al. (2015). We selected *Trifolium hybridum* as the host plant as it was found by Weese et al. (2015) to have the strongest detectable response to inoculation with the field-isolated rhizobium strains.

### Greenhouse study

We purchased *Trifolium hybridum* seeds from Ernst Conservation Seeds (Meadville, PA, USA). Prior to planting, seeds were washed and surface-sterilized in 90% ethanol, and soaked for 5 min in commercial bleach. Seeds were planted in media containing a 1:1:1 ratio of Berger BM6 All-Purpose Mix (Berger, Saint-Modeste, QC, Canada), torpedo sand, and Turface (Turface Athletics, Buffalo Grove, IL, USA). We chose this medium as it is relatively low in nitrogen. Prior to planting, the media was autoclaved at 121 °C for 30 min to sterilize and heavily rinsed to leach standing nitrogen.

Seeds were planted in 4-inch pots and grown in a complete randomized block design, with greenhouse bench as the blocking variable. Pots were split into eight treatments: six inoculated treatments corresponding to each rhizobium strain, an uninoculated treatment where nitrogen was added (referred to as the “N-addition treatment”), and an uninoculated control where no nitrogen was added. The experiment contained three blocks, with 96 pots per block consisting of 12 replicates of each treatment (*N* = 288). We inoculated all plants approximately 16 days after planting to ensure all seeds germinated and sprouted. Liquid cultures of each rhizobium strain were prepared in Yeast-Tryptone media for 36–48 h prior to inoculation and diluted to OD600 = 0.2. Inoculated plants received 500 µL of liquid culture of the appropriate rhizobium strain, while uninoculated plants (control and N-addition) received 450 µL of autoclaved Yeast-Tryptone media as a negative control. All plants in the N-addition treatment received 50 mL of 500 ppm NH4NO3 once a week for four weeks in December 2022, which amounted to a total of 12.3 g/m^2^ of nitrogen, equivalent to the field plot application at the Kellogg Biological Station LTER (Weese et al. 2015). Data from Weese et al. (2015) and pilot studies for this experiment indicated low growth in low-quality treatments, which we anticipated would limit the amount of plant material available to complete other components of this experiment. To address this, 12 additional plants were grown for treatments LQ651, LQ699, and the uninoculated control (*N* = 36, 12 per block) in the same greenhouse. Plants were grown under supplemental light on a 12:12 L: D photoperiod and watered twice daily between October 2022 and February 2023.

### Herbivore survival and development

*Spodoptera exigua* is a common generalist pest and a well-studied insect herbivore frequently used in studies of herbivory. Eggs were obtained from Benzon Research (Carlisle, PA, USA), and neonate larvae were hatched and reared on an artificial diet (Southland Products Inc., Lake Village, AR, USA) for approximately three days before the start of the experiment to reduce initial mortality. Following this, 240 instar larvae were weighed and transferred to individual 2-ounce plastic cups lined with filter paper. Each larva was assigned to one of eight feeding groups corresponding to each greenhouse treatment (*N* = 30 larvae per greenhouse treatment). Larvae were reared at 25 °C with a 14:10 L: D photoperiod. Throughout the experiment, larvae were given *ad libitum* access to leaves harvested from the greenhouse and allowed to eat to satiation. Leaves were harvested regularly, wrapped in damp paper towels, and stored in plastic bags at 1–4 °C prior to use. Due to low plant growth, particularly in the uninoculated control and LQ699 treatment, leaves were harvested from the same plants used for chemical and nutritional assays.

Survival and development of *S. exigua* were monitored daily. Molting was recorded when an exuvial skin was found in the larva’s container. Larval weight was measured for each instar approximately 24 h after molting. Upon pupation, development time (days to from hatching to eclosion), pupal weight, and sex were recorded. Following pupation, eclosion success was recorded, and adult *S. exigua* were euthanized, weighed, pinned, and spread. Overall survival rate was calculated as the number of individuals which survived to eclosion divided by the total number of individuals alive at the start of the experiment. All eclosed individuals were photographed and wing area was determined using ImageJ2 software (Fiji, Bethesda, MD, USA). 

### Plant measurements

To test whether any observed effects of rhizobia on herbivore survival and development (see Results) could be explained by plant nutritional traits or defensive compounds, we measured several plant characteristics. First, in January 2023, we measured plant height and chlorophyll content. Chlorophyll content, a common proxy measurement for plant nitrogen (Bullock and Anderson 1998; Swiader and Moore 2002), was measured using an SPAD-502 Plus chlorophyll meter (Spectrum Technologies, Aurora, IL, USA). Chlorophyll content was used over other direct nitrogen measurements as it is a non-destructive method (Gitelson et al. 2003) which would not deplete available plant material for other components of the experiment or induce defenses before plants were harvested later in the study. Additionally, we wanted our results to be comparable with Weese et al. (2015), which also used this method. After approximately 3 months of growth, we harvested plant leaves for tissue nutrition analysis. At least 120 mg worth of leaf tissue was collected from 2 randomly selected plants per block per treatment, for a total of six sampled plants per treatment. Immediately after collection, leaves were frozen in liquid nitrogen and stored at -80 °C. Leaf tissues were then freeze-dried in a lyophilizer (FreeZone 2.5 Plus, Labconco Corporation, Kansas City, MO, USA) and stored in a desiccator. Leaf tissues were pooled by treatment for analysis due to low growth in some treatments. Soluble protein content of leaf tissues was measured via Bradford Assay (Bradford 1976) using bovine gamma globulin as a protein standard. Digestible carbohydrate content was measured using a colorimetric assay defined by the protocol described in Deans et al. (2018) based on DuBois et al. (1956) and Smith et al. (1964). Spectrophotometry for either assay was completed using a Synergy HTX Mulitmode Absorbance Reader (BioTek, Winooski, VT, USA) and a NanoDrop 2000c Spectrophotometer (Thermo Fisher Scientific, Waltham, MA, USA), respectively. Measures of protein content and carbohydrate content were repeated in triplicate for each treatment (*N* = 24). We recorded soluble protein content and digestible carbohydrate content as percent soluble protein per milligram of dried plant material and percent carbohydrate per milligram of dried plant material, respectively. These measurements were used to calculate carbohydrate-to-protein ratio of each technical replicate.

### Plant metabolite analysis

In February 2023, we harvested remaining plant leaves for metabolomic analysis. Leaves were cut from the plant, frozen in liquid nitrogen, and stored at -80 °C. Collected material was pooled together by treatment and separated into five technical replicates per treatment, except for the unfertilized control and LQ699 treatments, for which only three and two replicates could be attained, respectively. Samples were sent to the Carver Metabolomics Core at the Roy J. Carver Biotechnology Center at the University of Illinois Urbana-Champaign for both targeted metabolomic analysis of the phytohormones jasmonic acid, salicylic acid, abscisic acid, and 12- oxo-phytodienoic acid (12-OPDA), as well as untargeted metabolomic analysis. Targeted analyses were performed using liquid chromatography-mass spectrometry (UHPLC-MS/MS). After adding 1 mL of methanol, the samples were then homogenized for 2–3 min using a bead mill. After centrifugation, 100 µL of supernatant was transferred to a vial pre-spiked with 5 µL of 5 µg/mL of jasmonic acid-d5, salicylic acid-13C6 and abscisic acid-d6. Extracts were analyzed with a 10 µL injection on an Agilent 1290 Infinity II UHPLC system (Agilent, Santa Clara, CA, USA), with an Agilent Poroshell 120 EC-C18 (1.9 μm, 2.1 × 50 mm) column at a column temperature of 35 °C and a flow rate of 300 µL/min. Mobile phases consisted of 0.1% formic acid in water and 0.1% formic acid in acetonitrile. Data was collected on a Sciex 5500 QTrap mass spectrometer (Sciex, Framingham, MA, USA). Data was acquired in negative mode MRM mode using Sciex Analyst v1.7.3. Peak integration and quantitation using calibration curves adjusted for internal standard were done using MultiQuant v3.1 software.

To perform untargeted metabolite analysis, plant samples were initially spiked with a mixture of deuterium and stable isotope labeled and non-labeled surrogate internal standards prior to processing. Processed samples were then analyzed using a Dionex Ultimate 3000 series UHPLC system (Thermo Scientific) with a Q-Exactive MS system (Thermo Scientific), as described by Bonini et al. (2020). A reversed phase liquid chromatography (RPLC) was performed using a Waters Acquity ethylene-bridged hybrid (BEH) C18 column (100 mm × 2.1 mm; 1.7 μm) column maintained at 25 °C with a flow rate of 0.3–0.4 mL/min (Waters Corp). The mobile phases consisted of water including 0.1% formic acid and solvent acetonitrile including 0.1% formic acid. Spectra were acquired in positive and negative ionization mode.

All LC-MS raw data files were analyzed using MS-DIAL ver.4.90 software for data collection, peak detection, alignment, adduct, and identification (Tsugawa et al. 2015). Compounds were annotated by m/z, MS/MS spectra, and retention time against an in-house library produced using chemical standards. In addition, they were annotated by m/z and MS/MS spectra using the public libraries’ MassBank of North America (MoNA), MassBank Europe (MassBank EU), and Global Natural Products Social Molecular Networking (GNPS), as well as the commercial library National Institute of Technology. Internal standards were monitored for retention time and intensity.

### Statistical analysis

All statistical analyses were performed in R 4.4.1 (R Core Team 2024). *Spodoptera exigua* survival over time as a function of treatment (6 rhizobium strains, uninoculated control, and fertilized) was analyzed via Cox proportional hazards regression (Cox 1972), using the “survival” and “rms” R packages (Therneau 2024; Harrell 2026). The regression model fit was tested using the proportional hazards assumption. Fisher’s LSD contrasts were used as a *post-hoc* test to determine differences between treatments (Supplemental Table 2). Developmental characters of *S. exigua*, including larval weight at each instar, development time, pupal weight, adult wing size, and adult dry mass, were analyzed via ANOVA with Tukey-Kramer HSD *post- hoc* tests. All variables met the assumptions for normality and homogeneity of variances.

**Fig. 1:**
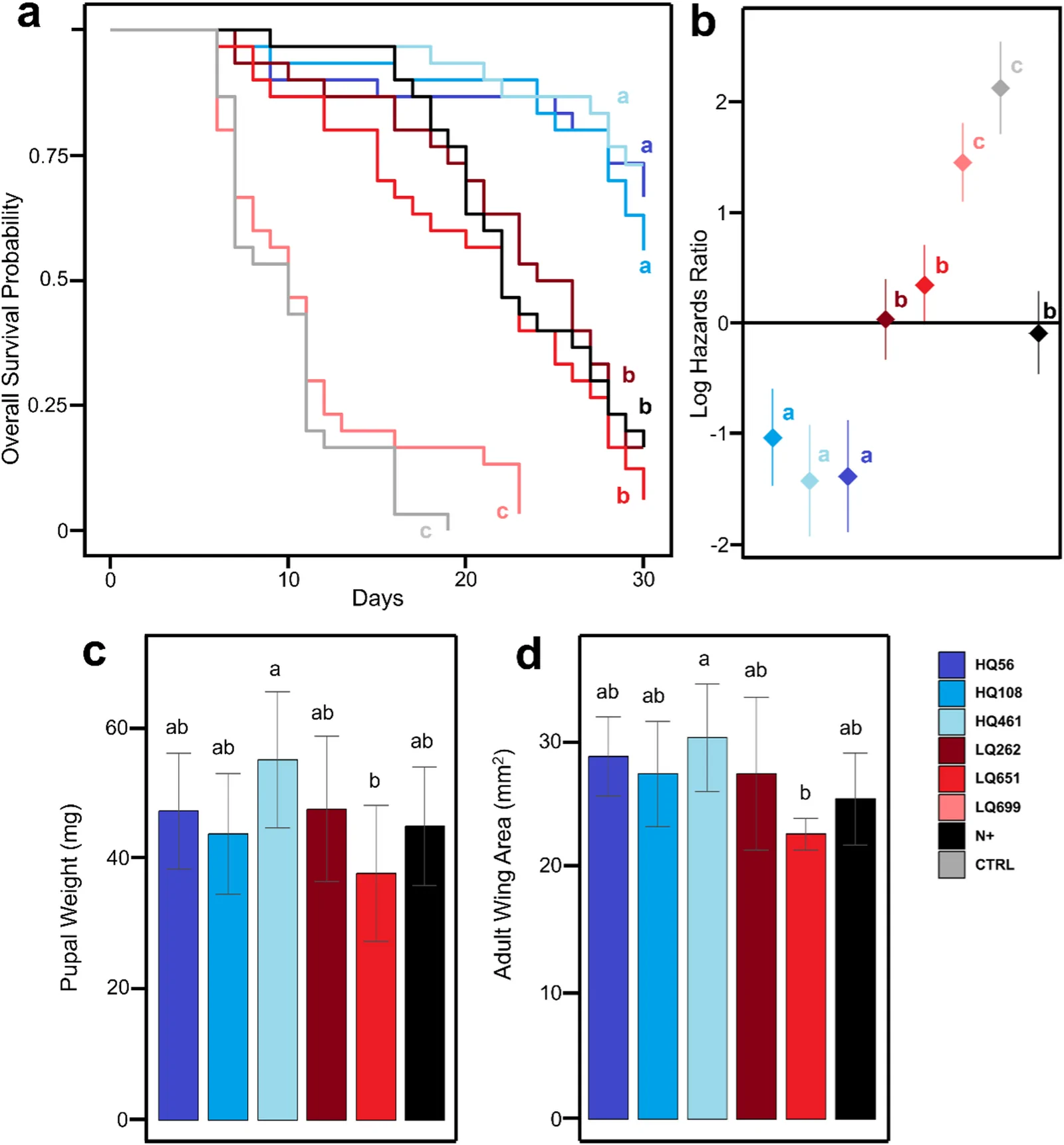
Survival and growth of *Spodoptera exigua.* (**a**) Kaplan-Meier survival curve. Letters indicate *post-hoc* results for survival analysis. (**b**) Log hazards ratio (± 95% CI) associated with partner quality. Values above the center line (positive) indicate higher probability that an individual will die on a given day; values below the center line (negative) indicate lower probability that an individual will die on that day. (**c**) Mean pupal weight (± SE) per treatment. (**d**) Mean adult wing area (± SE) per treatment

An analysis of variance (ANOVA) test and Tukey-Kramer HSD *post-hoc* test were performed to determine the effect of rhizobium treatment and greenhouse block on chlorophyll content and plant height. Treatment was included as a fixed factor and block as a random factor. All variables met the assumptions for normality and homogeneity of variances (Fig. 1).

**Fig. 2:**
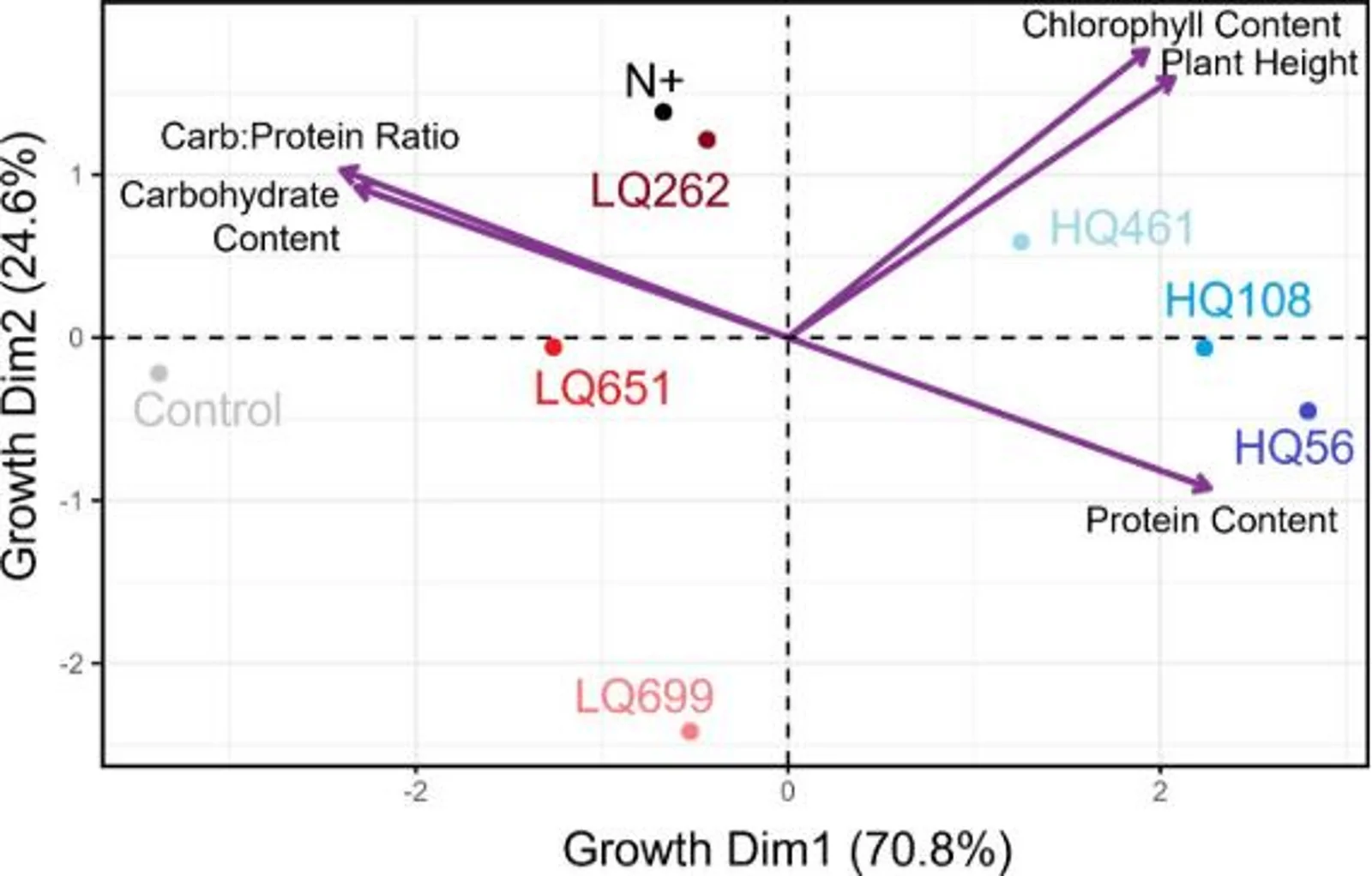
Plant nutrition principal component analysis (PCA) biplot showing relationships between *T. hybridum* nutrition variables and treatments. Purple arrows indicate plant measurements and their loadings in the PCA. Individual colored points indicate treatments

We conducted two principal component analyses (PCA) to assess how plant tissues from each treatment differed in terms of growth, nutrition, and defense; hereafter referred to as the “plant nutrition PCA” and the “plant metabolite PCA”. As a multivariate analysis method, PCA provides a comprehensive assessment of combined plant variables that could be affecting insect herbivore response. Both PCAs were performed using the “FactoMineR” R package (Lê et al. 2008), designed for multivariate exploratory data analysis. The first PCA, the plant nutrition PCA, summarized measurements for chlorophyll content, plant height, soluble protein content, digestible carbohydrate content, and carbohydrate-to-protein ratio based on treatment means. For treatments where chlorophyll content could not be measured due to low growth (treatment LQ699 and the uninoculated control), we estimated mean chlorophyll content using mean plant height, which was tightly correlated (R^2^= 0.98, *p* < 0.001) with chlorophyll content in the other treatments. Dimension 1 (hereafter referred to as Growth Dim1) of the plant nutrition PCA explained 50.3% of variation and Dimension 2 explained 21.9% of variation in herbivore response (Fig. 2). As this PCA utilized summarized data, we were unable to perform significance testing.

**Fig. 3:**
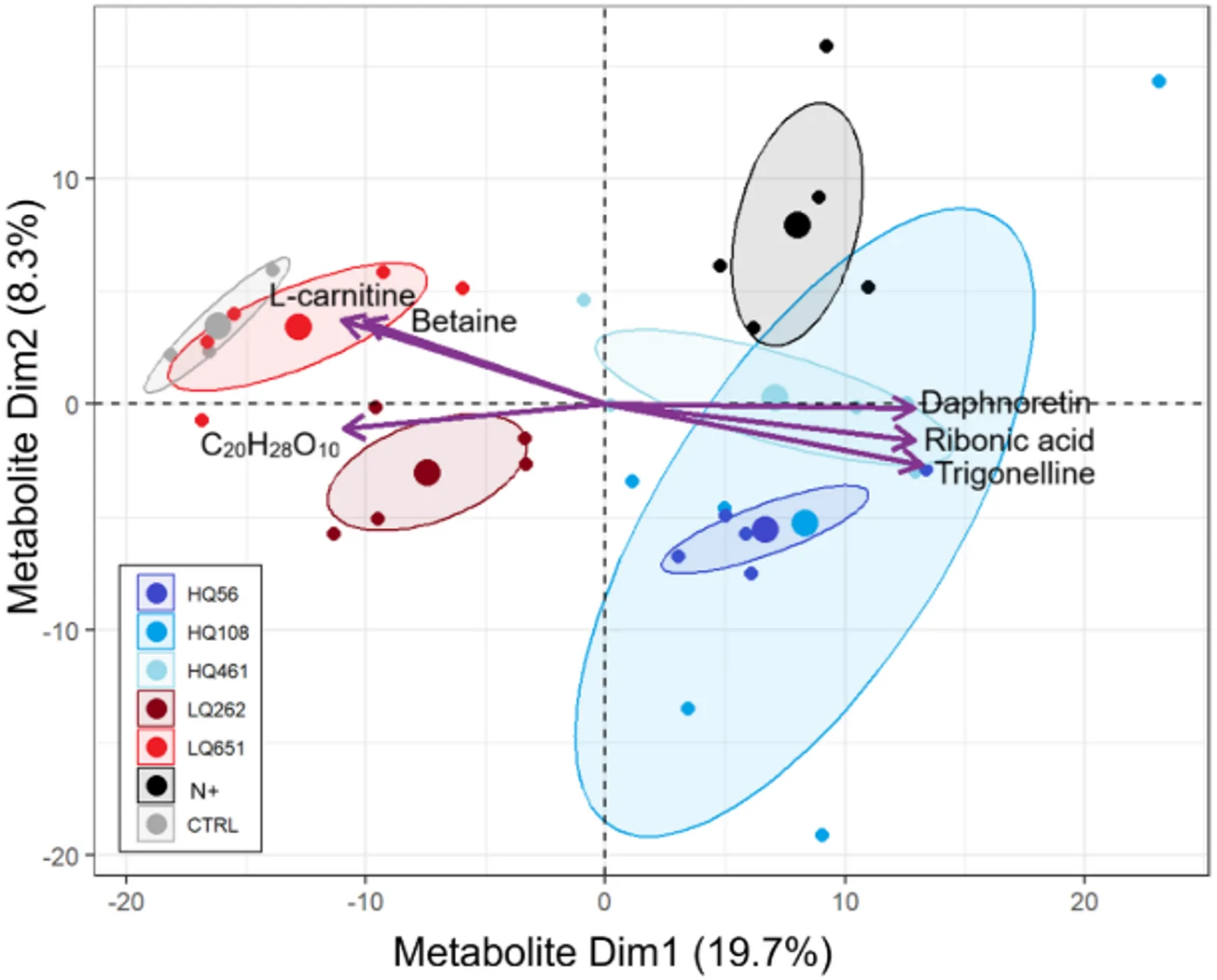
Plant metabolite principal component analysis (PCA) biplot visualizing metabolomic profiles of *T. hybridum* tissues from each experimental treatment. Large points represent means for each treatment group, while each small point represents a single technical replicate. Colored ellipses represent 95% CI for each treatment. Purple arrows indicate the compounds with the three highest loadings in the PCA (trigonelline, daphnoretin, and ribonic acid) as well as those with the three lowest loadings (L-carnitine, C_20_H_28_O_10_, and betaine)

The second PCA, the plant metabolite PCA, was analyzed separately from the plant nutrition PCA to better address plant chemistry. A large quantity of known metabolites was recovered by the Metabolomics Core (767 known metabolites); unknown metabolites found by the Metabolomics Core were excluded from all analyses. Prior to PCA, metabolite data was processed in MetaboAnalyst 6.0 (Pang et al. 2024), an online platform dedicated to metabolomics analysis. Processing included filtering the list of metabolites to exclude compounds based on low variance, which allows for the removal of metabolites which are constant across experimental conditions with the aim of eliminating baseline noise in the PCA (Hackstadt and Hess 2009). This resulted in the removal of 25% of compounds from the analysis, for a total of 575 compounds included in the PCA. Removed compounds included the four major plant phytohormones (jasmonic acid, salicylic acid, abscisic acid, and 12-OPDA), all of which had low variance and did not differ between treatments. Following this, all metabolite data was mean-centered to make compounds comparable in the analysis. Dimension 1 (hereafter referred to as Metabolite Dim1) of the plant metabolite PCA explained 19.8% of the variation and Dimension 2 explained 8.3% of the variation in metabolomic profiles (Fig. 3). Concentrations for the compounds with the three highest loading scores and three lowest loading scores in the plant metabolite PCA were identified and analyzed via ANOVA (Supplemental Fig. 2). To further evaluate the separation of treatment groups in the plant metabolite PCA, a PERMANOVA test was performed using the adonis2 function from the “vegan” R package (Oksanen et al. 2026), with further *post-hoc* testing performed using the “pairwiseAdonis” R package (Martinez 2020).

To compare herbivore response variables to plant traits, we extracted both Growth Dim1 and Metabolite Dim1 from the plant nutrition PCA and the plant metabolite PCA, respectively, and performed linear regressions against each herbivore response variable.

## Results

### Herbivore survival and development

Of the 240 *S. exigua* larvae hatched at the start of the experiment, 55 survived to eclosion. Of these surviving individuals, 82% belonged to a feeding group fed with a high-quality rhizobium treatment, 9% belonged to a group fed with a low-quality rhizobium treatment, and 9% belonged to a group fed with plants from the uninoculated N-addition treatment. All individuals in treatment LQ699 and the uninoculated, unfertilized control group died before pupation. Treatment had a highly significant effect on survival (Wald χ^2^ = 172.28, df = 7, *p* < 0.001), with strain LQ699 and the uninoculated, unfertilized control having the lowest survival probabilities (Fig. 1, Supplemental Table 2). Notably, strain LQ699 had lower survival probability than other low-quality strains, and the uninoculated N-addition treatment had lower survival probability than all high-quality strains (Fig. 1, Supplemental Table 2).

Treatments had varying effects on *S. exigua* larval weight, pupal weight, wing area, and adult dry mass. Larval weight in instars 3–5 was significantly affected by treatment (Instar 3: F_7,22_ = 3.94, *p* < 0.001 ; Instar 4: F_7,22_ = 3.99, *p* < 0.001; Instar 5: F_7,22_ = 2.5, *p* = 0.02) (Supplemental Fig. 1). During instars 3 and 4, caterpillars fed leaves from the N-addition control were heaviest; during the final instar, larval weights were highest in treatment HQ461 and lowest in treatment LQ651. In addition to larval weight, pupal weight (F_5,24_ = 4.02, *p* = 0.003) and adult dry mass (F_5,24_ = 3.56, *p* = 0.008) were affected by treatment. *Post-hoc* analysis revealed similar trends between these variables; *S. exigua* individuals in group HQ461 tended to have the largest pupal weights and greatest wing area of all other treatments (Fig. 1). Within low-quality treatment groups, individuals in group LQ262 tended to have higher pupal weight and larger wing areas compared to those in LQ651 (Fig. 1). Despite these results, there was no difference in development time or adult dry mass between treatments, and all caterpillars that pupated eventually eclosed. As no individuals fed leaves from the uninoculated, unfertilized control nor strain LQ699 survived to pupation, developmental data beyond larval weight could not be collected from *S. exigua* from these groups.

### Plant growth and metabolites

Rhizobium treatments affected both plant growth and plant metabolites. Due to low plant growth in the uninoculated control treatment and one of the low-quality strain treatments (LQ699), we were unable to accurately take chlorophyll data for all plants. The remaining treatments significantly affected chlorophyll content (F_5,30_ = 32.63, *p* < 0.001; Block: F_2,10_ = 4.78, *p* = 0.01). Plants inoculated with high-quality strains or fertilized with synthetic fertilizer had the highest chlorophyll content, while one of the low-quality strains resulted in substantially lower chlorophyl content (Supplemental Table 1). Similarly, plant height was highest in the high-quality and N-addition treatments compared to most low-quality treatments (F_7,29_ = 62.05, *p* < 0.001). Fertilized plants, plants inoculated with higher quality partners, and plants inoculated with strain LQ262 were taller than plants inoculated with low-quality strains LQ651 and LQ699 and control plants (Supplemental Table 1). Control plants and plants inoculated with strain LQ699 were much shorter than other plants in the experiment, and plant growth in these treatments remained low throughout the duration of the study.

In the plant nutrition PCA, high-quality treatments (HQ56, HQ108, HQ461) were separated from the low-quality treatments, the N-addition treatment, and the control along Growth Dim1, and one low-quality strain, LQ699, was separated from all other treatments along Growth Dim2 (Fig. 2). Protein content, chlorophyll content, and plant height, had high positive loadings on Growth Dim1, while carbohydrate content and carbohydrate-to-protein ratio had low negative loadings (Supplemental Table 3). In Growth Dim2, protein content had the sole negative loading compared to other plant variables.

In the plant metabolite PCA, there was again notable separation between low- and high- quality strains along Metabolite Dim1 (Fig. 3). There was overlap both between the N- addition treatment and the high-quality rhizobium treatments, as well as between the control and strain LQ651, indicating similarity in their metabolomic profiles. This separation of treatments was supported by further PERMANOVA, which revealed that all high-quality strains clustered together, and low-quality strains and the uninoculated control treatment clustered together (F6,26 = 4.596, R2 = 0.515, *p* < 0.001). The N-addition treatment clustered apart from all other treatments (Supplemental Table 4). However, when applying Bonferroni p-value adjustment to this data, it became insignificant due to low sample size among treatments. The plant-derived compounds with the three highest scores for Metabolite Dim1 included trigonelline (x = 0.878), daphnoretin (x = 0.859), and ribonic acid (x = 0.859), while those with the three lowest scores included L- carnitine (x =-0.731), 2-phenylethyl 3-O-(4-carboxy-3-hydroxy-3-methylbutanoyl)-β-D- glucopyranoside (referred hereafter by its molecular formula, C20H28O10) (x =-0.728), and betaine (x =-0.668) (Supplemental Table 5). Jasmonic acid, salicylic acid, abscisic acid, and 12- OPDA, phytohormones relevant to plant defense which we had targeted in our metabolite analyses, did not differ between treatments and were screened out during processing of metabolite data for the PCA.

### Herbivore response to plant measurements

**Fig. 4:**
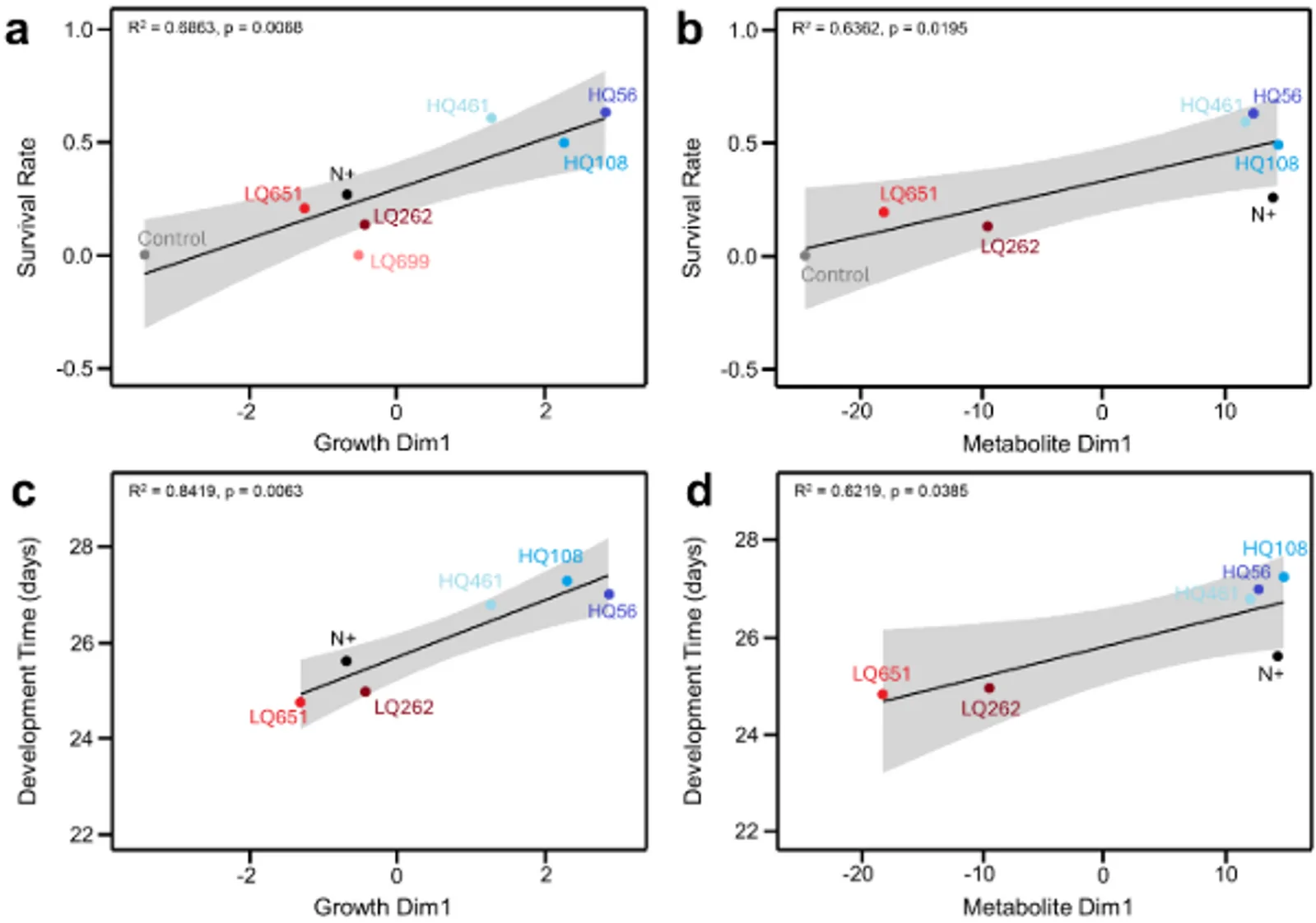
Linear regressions of *S. exigua* (**a**, **b**) survival rate and (**c**, **d**) development time (days from hatching until eclosion) against (**a**, **c**) the most explanatory PCA dimension in the plant nutrition PCA, Growth Dim1 and (**b**, **d**) the most explanatory PCA dimension in the plant metabolite PCA, Metabolite Dim1. Gray areas represent 95% CI for each model. Colored dots correspond to rhizobium treatment. Some values for certain groups, particularly LQ699 and the uninoculated control, are missing due to exclusion from PCAs

The effects of rhizobia and N treatments on herbivore survival and development metrics might be explained in part by rhizobium effects on plant leaf characteristics and metabolites. First, both Growth Dim 1 and Metabolite Dim 1 were positively correlated with *S. exigua* survival rate (Growth: R^2^ = 0.69, *p* = 0.007; Metabolite: R^2^ = 0.64, *p* = 0.02) and development time (Growth: R^2^ = 0.84, *p* = 0.006; Metabolite: R^2^ = 0.62, *p* = 0.039) (Fig. 4), but not other developmental metrics. Growth Dim 1 and Metabolite Dim 1 were also positively correlated with each other (R^2^ = 0.63, *p* = 0.021).

## Discussion

Plant-associated microbial mutualists are strong predictors of plant growth and can affect higher trophic levels by altering plant traits (Koricheva et al. 2009). While existing work in this field has focused on how variation in quality among mutualists can impact herbivore damage or presence (Dean et al. 2009; Simonsen and Stinchcombe 2014), here we show that mutualist variation can also impact the survival and development of a generalist insect herbivore, *Spodoptera exigua*. Notably, *S. exigua* fed leaves from plants inoculated with high-quality rhizobium partners had higher survival rates and increased body size compared to those fed leaves from other treatments, including plants treated with synthetic nitrogen fertilizers. This work demonstrates that beyond the presence of microbial resource mutualists, genetic variation in mutualist quality strongly affects herbivores and that synthetic nitrogen does not replace the benefits of high-quality rhizobia for herbivore survival and growth. It should be noted that, while we compensated for limited plant biomass among low-growth treatments in this study, in a natural setting, plants associated with low-quality mutualists will likely not provide sufficient volume of forage for herbivores due to low growth. As such, this study focused primarily on the quality of forage provided by plants associated with variable-quality mutualists, rather than accounting for how available biomass may impact herbivore response.

The observed differences in *S. exigua* growth across strains may be directly linked to the genetic variability in rhizobia mutualists and their impacts on nitrogen availability in plants. Past research has found that inoculation of a host plant with a single rhizobium strain can influence herbivore responses via increasing plant quantity and nutrition as well as plant defenses (Throop and Lerdau 2004; Edwards and Singh 2006; Ballhorn et al. 2013; Dean et al. 2014). When taking multiple strains of varying mutualist quality into account, there is potential for great variability in plant foliar traits, as N-fixing rhizobia control a leguminous plant’s access to N, even in N-poor soils (Parker 1995; Weese et al. 2015; Seufert et al. 2019). Nitrogen limitation can decrease rates of photosynthesis through declines in chlorophyll content and electron transport chain efficiency (Aerts et al. 1995; Zhou et al. 2006; Peng et al. 2021), and can impact foliar traits by decreasing leaf area expansion and increasing starch accumulation in leaf tissues (Seufert et al. 2019). Limited nitrogen can further decrease amounts of amino acids and nitrogen-containing compounds, impairing plant defenses against environmental stress and herbivore attack (Zhou et al. 2021). Ultimately, nitrogen availability, mediated by the quality of the rhizobium mutualist, is the likely mechanism mediating herbivore response in this study, as reflected in differences in chlorophyll content, plant height, general plant nutrition, and plant metabolomics.

While fertilizers are expected to produce higher quality forage via increasing foliar N, which in turn increases herbivore performance (Chen et al. 2008; Wang et al. 2018), in our study, fertilizing plants with synthetic nitrogen did not restore *S. exigua* survival. This suggests that synthetic nitrogen deposition, while generally increasing aboveground plant biomass, does not increase the quality of forage to the degree that high-quality rhizobium mutualists do, at least in this particular system. In legumes, synthetic fertilizer application typically achieves similar levels of photosynthesis as inoculation with rhizobia (Zhou et al. 2006), and we found similar amounts of chlorophyll content in our N-addition treatment and our high-quality treatments. Despite this, the N-addition treatment aligned more closely with high-quality treatments in our plant metabolite PCA but aligned more closely with the low-quality treatments in our nutrition PCA, suggesting that the differences in survival for caterpillars in the high-quality vs. N-addition treatment may be explained by nutrition rather than chemistry. While legumes generally have low carbon-to-nitrogen (C: N) ratios compared to other plants, Heath et al. (2020) found that variation in C: N ratio within species can depend on rhizobium partner quality, with higher C: N ratios in plants associated with lower quality rhizobia. Similar to plants associating with lower-quality mutualists, in our study plants in the N-addition treatment had higher carbohydrate-to-protein ratios, likely due to the absence of resource exchange. A high carbohydrate-to-protein ratio in N-addition leaf tissues might not be adequate to support the nutritional requirements of *S. exigua*, which have been known to have the highest survival rates when fed equal C: N ratio diets (Ma et al. 2023; Zhang et al. 2026). Insect feeding behavior studies show that high-sugar diets in *Drosophila melanogaster* have been shown to produce more reactive oxygen species in insect tissues, disrupting homeostasis (Strilbytska et al. 2022; Huang et al. 2024). Thus, while plants in the N-addition treatment contained similar amounts of chlorophyll content, an imbalanced C: N ratio in leaf tissues could have increased nutritional stress in the caterpillars, resulting in decreased survival.

Both in our study and past research, rhizobium strain affected plant foliar N in the form of chlorophyll content (Weese et al. 2015), and our plant nutrition PCA results further show that nutrition broadly differs between low-quality and high-quality strains. Protein and carbohydrate content of plant leaves had strong loadings along Growth Dim1, indicating that differences in either macromolecule contributed to differences between strains. The correlation of herbivore survival and development time with Growth Dim1 further suggests the role of nutrition impacting insect response. This is unsurprising, as nutrient content of plant diets is a primary driver of insect herbivore survival and development (Mattson 1980; Scriber and Slansky 1981; Audusseau et al. 2015; Pöyry et al. 2017). Defenses and phytochemistry, however, can also play a role in insect response, as plant defenses are frequently upregulated by association with rhizobia (Edwards and Singh 2006, Dean et al. 2014). In the plant metabolite PCA, low-quality and high-quality strains were again separated from each other. Trigonelline, daphnoretin, and ribonic acid were the top positive scoring compounds in our analysis. Trigonelline and daphnoretin are both defensive compounds found broadly across plant families (Rehman et al. 2012; Kicel and Wolbis 2012; Dillon et al. 2024), while ribonic acid is a ribose derivative which has been shown to increase in plants when they are inoculated with beneficial soil microbes (Schillaci et al. 2021). L-carnitine and betaine, two of the lowest-scoring compounds, are both associated broadly with plant stress management (Jacques et al. 2018; Oney-Birol 2019; Li et al. 2025). Plant-derived carnitine in particular can be affected by belowground mutualists such as arbuscular mycorrhizal fungi (AMF), and increased carnitine in insect tissues has been linked to an increase in deformities in *Manduca sexta* (Papantoniou et al. 2021). The functions of these metabolites provide a general idea of what types of compounds may be driving *S. exigua* response, although it is likely the whole plant metabolome, mediated by N-limitation via mutualist quality, could be contributing to responses, as found in other multi-trophic plant metabolite studies (Arany et al. 2008; Leiss et al. 2009; Kuzina et al. 2009; Badri et al. 2013; Huberty et al. 2022; Yuan et al. 2023). While untargeted metabolite analysis has been criticized for being correlative and not causative (Maag et al. 2015), these results highlight particular plant features that can be targeted in future mechanistic studies.

The clear separation of low-quality strains from high-quality strains in both the plant nutrition PCA and the plant metabolite PCA shows that these strains were distinct in terms of both nutrition and phytochemistry. Taken in concert, it is evident that high-quality rhizobia mutualists produce higher quality plants in terms of both nutrition and, based off the influence of defensive compounds in the plant metabolite PCA, secondary defenses. These findings are consistent with previous research evaluating herbivore response to mutualist variation, given that beneficial microbes are more likely to promote plant growth, which in turn is associated with greater herbivore preference (Simonsen and Stinchcombe 2014). While secondary defenses typically decrease herbivore abundance and survival (Thamer et al. 2011; Basu et al. 2022; Dillon et al. 2024), legumes with elevated defensive compounds sometimes have positive effects on herbivores in lab settings (Simpson and Simpson 1990; Dean et al. 2014; Bustos-Segura et al. 2024). We suspect that, for *S. exigua*, the overall quality of their host plant outweighs the potential costs of plant defenses, similar to what has been hypothesized in past research (Dean et al. 2014; Bustos-Segura et al. 2024). This is supported by the evidence that, although Growth Dim1 and Metabolite Dim1 from our PCAs were both positively correlated with *S. exigua* survival, they were also positively correlated with development time, indicating a possible tradeoff in response. While better nutrition in the high-quality treatments helped to improve *S. exigua* survival, enhanced defenses could have delayed caterpillar development as resources used for growth were re-allocated to the detoxification of plant secondary metabolites. Despite this, *S. exigua* fed plants inoculated with some high-quality strains had greater pupal mass and wing area, also suggesting that defenses, while slowing development time, may not have impacted caterpillar physiology enough to reduce body size. There is apparent complexity in herbivore responses to mutualist variability and the ecological outcome of these multi-trophic interactions would likely be influenced by factors beyond the scope of this experiment. In real-world contexts, longer development times and larger body size could influence herbivore survival by increasing risk of attack by natural enemies as proposed by the slow growth-high mortality hypothesis (Chen and Chen 2018), indicating an additional ecological tradeoff of feeding on plants with high-quality mutualists. While our results show that herbivores may favor increased nutritional quality of plant forage over digestibility, plants associating with high-quality rhizobia partners may benefit from top-down regulation of herbivory over those associated with low-quality partners.

In sum, this lab-based study demonstrates that variation in rhizobia can scale up to affect higher trophic levels. While these findings may not directly translate to the field, they expand upon work in the realm of community genetics demonstrating how plant genetic variation influences herbivore and predator communities (reviewed in Crutsinger 2016) by showing that genetic variation in belowground microbial symbionts scales up to affect plant phenotypes (“extended phenotypes” of rhizobia) in ways that affect higher trophic levels. Interestingly, the effects of high-quality rhizobia are not just due to increased nitrogen availability as synthetic fertilizers do not benefit herbivore survival and growth to the same extent. Instead, high-quality rhizobia appear to have effects on both plant nutrition and metabolites that differ from the effects of synthetic nitrogen on plant chemistry, resulting in rhizobia effects on higher trophic levels that cannot be recapitulated by synthetic fertilizers.

## Supplementary Information

Below is the link to the electronic supplementary material.

Supplementary Material 1

Supplementary Material 2

## Data Availability

Data will be deposited in the Illinois Data Bank and assigned a stable identifier for easy reference and citation.
