# Supplementary material for: Less cooperative belowground plant mutualists negatively affect aboveground herbivore growth and survival
Source: Oecologia. 2026 Sep 4;208(10):126. doi: 10.1007/s00442-026-05966-7 (PMC13545029; doi:10.1007/s00442-026-05966-7)
Supplement: Supplementary file 1 [file 442_2026_5966_MOESM1_ESM.docx]

**Supplemental Material 1**

**Less cooperative belowground plant mutualists negatively affect aboveground herbivore growth and survival**

Annaliese H. Wargin^1*^, Katy D. Heath^2^, Jennifer A. Lau^3^, Adam G. Dolezal^1^, Alexandra N. Harmon-Threatt^1^

^1^Department of Entomology, University of Illinois Urbana-Champaign, IL, USA, 61820

^2^Department of Plant Biology, University of Illinois Urbana-Champaign, IL, USA, 61820

^3^Department of Biology, Indiana University, IN, USA, 47405

^*^Corresponding author, [awargin2@illinois.edu](mailto:awargin2@illinois.edu)

Supplemental Material 1

**Supplemental Table 1**. Mean plant response measurements for each of the R*hizobium leguminosarum* strains, as recorded in this study, as well as reported in Weese *et al*. 2015. All means are reported for *Trifolium hybridum*.

| Strain Treatment | Chlorophyll content (nmol/cm^2^) (present study) | Plant Height (present study) | Soluble protein content per mg dry mass (%) | Digestible carbohydrate content per mg dry mass (%) | Chlorophyll Content (nmol/cm^2^)  (Weese et al. 2015) | Leaf Number (Weese et al. 2015) | Nodule Number  (Weese et al. 2015) |
| --- | --- | --- | --- | --- | --- | --- | --- |
| HQ56 | 44.64 | 11.40 | 17.38 | 30.96 | 46.82 | 15.6 | 32.8 |
| HQ108 | 45.20 | 11.20 | 17.04 | 36.10 | 48.8 | 40 | 259 |
| HQ461 | 46.15 | 10.71 | 15.72 | 42.19 | 45.975 | 27 | 124.25 |
| LQ262 | 42.98 | 9.28 | 12.09 | 43.50 | 18.567 | 6 | 154.6 |
| LQ651 | 28.12 | 5.91 | 14.18 | 50.96 | 20.16 | 9.5 | 401.33 |
| LQ699 | NA | 1.58 | 14.73 | 36.41 | 24.33 | 8.33 | 226.67 |
| Control | NA | 2.02 | 12.80 | 48.79 | 36.35 | 14.19 | 128.34 |
| N+ | 42.68 | 9.43 | 12.29 | 57.93 | NA | NA | NA |

**Supplemental Table 2**. Fischer’s LSD contrasts for *Spodoptera exigua* survival probability by treatment. Contrasts ± 1 SE are reported. Significant differences between the two pairwise groups are bolded. Smaller values indicate that the survival probability of the group listed first in the comparison is larger than that of the group listed second. Larger values indicate the survival probability of the second group in the comparison is smaller than that of the first.

|  | Pairwise Comparison | Contrast |
| --- | --- | --- |
| Within HQ strains | HQ56 / HQ108 | -0.348 ± 0.364 |
|  | HQ56 / HQ461 | 0.044 ± 0.393 |
|  | HQ108 / HQ461 | 0.392 ± 0.364 |
| Within LQ strains | LQ262 / LQ651 | -0.311 ± 0.271 |
|  | LQ262 / LQ699 | **-1.416 ± 0.272** |
|  | LQ651 / LQ699 | **-1.105 ± 0.269** |
| HQ vs LQ | LQ56 / LQ262 | **-1.416 ± 0.342** |
|  | LQ56 / LQ651 | **-1.726 ± 0.344** |
|  | LQ56 / LQ699 | **-2.832 ± 0.344** |
|  | LQ108 / LQ262 | **-1.068 ± 0.309** |
|  | LQ108 / LQ651 | **-1.378 ± 0.310** |
|  | LQ108 / LQ699 | **-2.484 ± 0.311** |
|  | LQ461 / LQ262 | **-1.460 ± 0.343** |
|  | LQ461 / LQ651 | **-1.77 ± 0.344** |
|  | LQ461 / LQ699 | **-2.876 ± 0.345** |

| Strains vs. Nitrogen+ | N+ / HQ56 | **1.292 ± 0.345** |
| --- | --- | --- |
|  | N+ / HQ108 | **0.944 ± 0.312** |
|  | N+ / HQ461 | **1.336 ± 0.345** |
|  | N+ / LQ262 | -0.123 ± 0.279 |
|  | N+ / LQ651 | -0.434 ± 0.278 |
|  | N+ / LQ699 | **-1.539 ± 0.278** |
| Strains vs. Nitrogen- | N- / HQ56 | **3.508 ± 0.371** |
|  | N- / HQ108 | **-3.160 ± 0.340** |
|  | N- / HQ461 | **3.551 ± 0.371** |
|  | N- / LQ262 | **2.092 ± 0.298** |
|  | N- / LQ651 | **1.781 ± 0.291** |
|  | N- / LQ699 | **0.676 ± 0.271** |
| N+ vs. N- | N+ / N- | **2.216 ± 0.303** |

**Table 2 (cont.)**

**Supplemental Figure 1**: Mean larval weight at each of the five larval instars and pupal stage. Points and whiskers indicate weight means ± SE for each treatment. Lines connect individual points to show the trajectory of growth over time.

**Supplemental Table 3**. PCA loadings for variables included in the plant growth and nutrition PCA for the first five dimensions of the PCA.

|  | Dim.1 | Dim.2 | Dim.3 | Dim.4 | Dim.5 |
| --- | --- | --- | --- | --- | --- |
| Chlorophyll Content | 0.736308 | 0.674568 | -0.03844 | -0.03394 | 0.013412 |
| Plant Height | 0.790652 | 0.609697 | 0.041848 | 0.034357 | -0.01439 |
| Carb-Protein Ratio | -0.91677 | 0.393015 | 0.055483 | 0.042395 | 0.014156 |
| Carbohydrate Content | -0.88666 | 0.353335 | 0.296613 | -0.03056 | -0.00853 |
| Protein Content | 0.864528 | -0.35297 | 0.357512 | 0.011102 | 0.008003 |

**Supplemental Figure 2**: Concentrations of the compounds which had the three highest loading scores in the plant metabolite PCA, trigonelline (A), daphnoretin (B), and ribonic acid (C), and the compounds which had the three lowest loading scores, L-carnitine (D), betaine (E), and C_20_H_28_O_10_ (F). Includes ANOVA results evaluating differences in concentration between each treatment.

Supplemental Table 4. PERMANOVA *post-hoc* results for significance testing of plant metabolite PCA. Significant results are asterisked and bolded. Marginally significant results are marked with a period.

| Pair | df | Sums of Squares | F-stat | R^2^ | p-value | p-value adjusted |
| --- | --- | --- | --- | --- | --- | --- |
| Control vs HQ56 | 1 | 1385.124 | 9.315 | 0.608 | **0.023*** | 0.483 |
| Control vs HQ108 | 1 | 1287.0445 | 4.361 | 0.421 | **0.022*** | 0.462 |
| Control vs HQ461 | 1 | 1054.135 | 7.059 | 0.541 | **0.018*** | 0.378 |
| Control vs LQ262 | 1 | 296.753 | 2.932 | 0.328 | 0.054. | 1.000 |
| Control vs LQ651 | 1 | 45.954 | 0.278 | 0.044 | 0.862 | 1.000 |
| Control vs N+ | 1 | 1321.337 | 7.482 | 0.555 | **0.010*** | 0.210 |
| N+ vs HQ56 | 1 | 1092.573 | 6.806 | 0.460 | **0.003*** | 0.063 |
| N+ vs HQ108 | 1 | 634.595 | 2.347 | 0.227 | 0.056. | 1.000 |
| N+ vs HQ461 | 1 | 357.833 | 2.222 | 0.217 | 0.054. | 1.000 |
| N+ vs LQ262 | 1 | 995.905 | 7.972 | 0.499 | **0.013*** | 0.273 |
| N+ vs LQ651 | 1 | 1415.675 | 8.179 | 0.506 | **0.007*** | 0.147 |
| HQ56 vs HQ108 | 1 | 384.556 | 1.542 | 0.162 | 0.242 | 1.000 |
| HQ56 vs HQ461 | 1 | 288.646 | 2.061 | 0.205 | 0.115 | 1.000 |
| HQ108 vs HQ461 | 1 | 134.851 | 0.540 | 0.063 | 0.679 | 1.000 |
| LQ262 vs LQ651 | 1 | 273.397 | 2.346 | 0.227 | 0.097 | 1.000 |
| HQ56 vs LQ262 | 1 | 980.045 | 9.424 | 0.541 | **0.012*** | 0.252 |
| HQ56 vs LQ651 | 1 | 1340.491 | 8.809 | 0.524 | **0.009*** | 0.189 |
| HQ108 vs LQ262 | 1 | 668.651 | 3.127 | 0.281 | **0.014*** | 0.294 |
| HQ108 vs LQ651 | 1 | 1342.330 | 5.12 | 0.390 | **0.009*** | 0.189 |
| HQ461 vs LQ262 | 1 | 664.609 | 6.361 | 0.443 | **0.008*** | 0.168 |
| HQ461 vs LQ651 | 1 | 1037.875 | 6.799 | 0.459 | **0.005*** | 0.105 |
