# Supplementary material for: Less cooperative belowground plant mutualists negatively affect aboveground herbivore growth and survival
Source: Oecologia. 2026 Sep 4;208(10):126. doi: 10.1007/s00442-026-05966-7 (PMC13545029; doi:10.1007/s00442-026-05966-7)
Supplement: Supplementary file 2 [file 442_2026_5966_MOESM2_ESM.docx]

**Supplemental Material 2**

**Less cooperative belowground plant mutualists negatively affect aboveground herbivore growth and survival**

Annaliese H. Wargin^1*^, Katy D. Heath^2^, Jennifer A. Lau^3^, Adam G. Dolezal^1^, Alexandra N. Harmon-Threatt^1^

^1^Department of Entomology, University of Illinois Urbana-Champaign, IL, USA, 61820

^2^Department of Plant Biology, University of Illinois Urbana-Champaign, IL, USA, 61820

^3^Department of Biology, Indiana University, IN, USA, 47405

^*^Corresponding author, [awargin2@illinois.edu](mailto:awargin2@illinois.edu)

Supplemental Material 2

**Supplemental Table 5**. PCA loadings for metabolites included in the plant metabolite PCA. Plant metabolite PCA contributions. Loadings have been sorted based on their value in Metabolite Dim1, the most explanatory PCA dimension.

|  | Dim.1 | Dim.2 | Dim.3 | Dim.4 | Dim.5 |
| --- | --- | --- | --- | --- | --- |
| TRIGONELLINE | 0.878538 | -0.19903 | 0.084345 | 0.125002 | -0.1531 |
| ETHYL HYDROGEN MALONATE | 0.878137 | 0.100595 | 0.009184 | -0.18453 | -0.05292 |
| UROCANATE | 0.876567 | -0.20661 | 0.078857 | 0.130079 | -0.16407 |
| DAPHNORETIN | 0.859266 | -0.03452 | 0.086245 | -0.20963 | -0.05407 |
| RIBONIC ACID | 0.858528 | -0.07487 | -0.20501 | 0.189595 | -0.2088 |
| CYTIDINE | 0.846697 | -0.17022 | 0.122644 | -0.00375 | -0.20809 |
| PICOLINIC ACID | 0.833641 | -0.0971 | 0.003023 | 0.105509 | -0.21443 |
| CYTOSINE | 0.828797 | -0.07835 | 0.065292 | 0.251147 | -0.1019 |
| GUANINE | 0.820283 | -0.02631 | 0.113136 | 0.268812 | -0.11915 |
| DEOXYADENOSINE | 0.816063 | -0.27541 | 0.175952 | 0.128628 | -0.11864 |
| CIS-4,7,10,13,16,19-DOCOSAHEXAENOIC ACID | 0.80889 | -0.11553 | 0.324119 | 0.058007 | 0.112677 |
| 1,5-ANHYDRO-1-(2-(3,4-DIHYDROXYPHENYL)-5,7-DIHYDROXY-4-OXO-4H-CHROMEN-6-YL)HEXITOL | 0.802956 | -0.04437 | 0.01769 | -0.26939 | -0.01956 |
| ASPARTATE | 0.788259 | -0.0299 | 0.085812 | -0.13609 | -0.15135 |
| DL-2,3-DIAMINOPROPIONIC ACID | 0.785801 | -0.15299 | -0.09676 | 0.066568 | 0.015303 |
| 3-(2-(BETA-D-GLUCOPYRANOSYLOXY)-3-HYDROXYPHENYL)PROPANOIC ACID | 0.779686 | -0.14764 | -0.02562 | -0.14968 | 0.001036 |
| 2-(4-HYDROXY-7-METHYL-5-OXO-2,3-DIHYDRO-5H-FURO[3,2-G]CHROMEN-2-YL)PROPAN-2-YL HEXOPYRANOSIDE | 0.774692 | 0.342964 | -0.34249 | 0.015752 | -0.16985 |
| 3-DEOXY-D-GLYCERO-D-GALACTO-2-NONULOSONIC ACID | 0.769968 | -0.10654 | -0.13797 | 0.438151 | -0.2142 |
| PHEOPHORBIDE A | 0.762967 | 0.247232 | -0.07877 | 0.000468 | 0.078666 |
| 7,8-DIHYDRO-ALPHA-IONONE | 0.758439 | -0.04993 | 0.352716 | 0.065529 | 0.174968 |
| HYDROXYPHENYLLACTIC ACID | 0.757821 | -0.18681 | 0.037442 | -0.24749 | -0.02395 |
| 3-METHYLGLUTACONIC ACID | 0.750373 | -0.0992 | 0.164489 | 0.118261 | 0.199264 |
| METHYL 4,6-O-BENZYLIDENE-ALPHA-D-GLUCOPYRANOSIDE | 0.749656 | 0.370938 | -0.3088 | 0.088363 | -0.16566 |
| PINOCEMBRIN | 0.748242 | -0.06271 | 0.156962 | -0.40274 | -0.10775 |
| GLUCONATE | 0.742728 | 0.280596 | 0.105269 | 0.13912 | -0.14994 |
| ECHINOCYSTIC ACID | 0.741061 | -0.15882 | 0.385193 | 0.073429 | 0.142071 |
| MYO-INOSITOL | 0.738928 | -0.03801 | 0.030224 | 0.451132 | -0.04486 |
| 3,4-DIHYDROXYHYDROCINNAMIC ACID | 0.73828 | -0.11643 | 0.003905 | -0.20484 | -0.031 |
| 4,5-DIMETHYL-1,3-BENZENEDIOL | 0.736198 | -0.18786 | 0.039552 | -0.26707 | -0.02444 |
| ANEMOSAPOGENIN | 0.735765 | -0.15188 | 0.360716 | 0.074147 | 0.153711 |
| 5-(HYDROXYMETHYL)-2-FURALDEHYDE | 0.733636 | -0.33482 | 0.003892 | -0.03655 | 0.104214 |
| HEDERAGENIN | 0.733342 | -0.18102 | 0.376524 | 0.083964 | 0.120378 |
| ASPARAGINE | 0.732135 | -0.18718 | -0.16787 | -0.22631 | -0.08831 |
| D-PINITOL | 0.731463 | -0.29635 | 0.016677 | -0.08529 | 0.037214 |
| (1R)-(-)-NOPOL | 0.731438 | -0.06706 | 0.351052 | 0.247421 | 0.120505 |
| (1S,4AR,5S)-5-[(E)-5-METHOXY-3-METHYL-5-OXOPENT-3-ENYL]-1,4A-DIMETHYL-6-METHYLIDENE-3,4,5,7,8,8A-HEXAHYDRO-2H-NAPHTHALENE-1-CARBOXYLIC ACID | 0.72969 | -0.18394 | 0.371853 | 0.111083 | 0.129052 |
| (-)-QUEBRACHITOL | 0.729266 | -0.37557 | 0.013972 | -0.02421 | 0.13126 |
| 2-ANTHRACENESULFONIC ACID | 0.728958 | -0.04231 | 0.101309 | 0.550265 | -0.03191 |
| 15ALPHA-HYDROXYCULMORIN | 0.728136 | -0.12956 | 0.344142 | 0.122423 | 0.177163 |
| URIDINE | 0.727392 | 0.028793 | 0.320688 | 0.207772 | -0.16359 |
| (3BETA,5XI,9XI,18XI)-23,28-DIHYDROXY-28-OXOOLEAN-12-EN-3-YL BETA-D-GLUCOPYRANOSIDURONIC ACID | 0.727152 | -0.13838 | 0.386411 | 0.040487 | 0.14321 |
| 4-DEOXYPHLORIDZIN | 0.726235 | 0.36954 | -0.2186 | -0.02292 | -0.25467 |
| THYMIDINE | 0.725224 | -0.23361 | 0.27219 | 0.084941 | -0.10929 |
| 2-KETO-3-DEOXYOCTONIC ACID | 0.72461 | -0.21367 | -0.09258 | 0.256988 | -0.0123 |
| BETA-ALANINE | 0.723502 | 0.031305 | 0.155112 | -0.04727 | -0.14632 |
| AFZELIN | 0.721449 | 0.212699 | -0.13836 | -0.31119 | 0.195074 |
| ALISOL B ACETATE | 0.721314 | -0.24693 | 0.352566 | 0.036105 | 0.168653 |
| 4,6-DIHYDROXYPYRIMIDINE | 0.719491 | -0.17793 | -0.18251 | -0.25084 | -0.06889 |
| (-)-CARYOPHYLLENE OXIDE | 0.718295 | -0.09969 | 0.37599 | 0.150871 | 0.143132 |
| N6-METHYLADENOSINE | 0.718106 | 0.20593 | -0.14669 | 0.391774 | -0.28737 |
| OLEANOLIC ACID | 0.710326 | -0.37177 | 0.135807 | 0.011516 | 0.168729 |
| MAESOPSIN | 0.708434 | 0.000365 | -0.22057 | -0.29472 | -0.155 |
| HOMOGENTISIC ACID | 0.707768 | -0.0995 | 0.002019 | -0.1968 | -0.03894 |
| (3E)-4-(2,4,6-TRIMETHYL-3-CYCLOHEXEN-1-YL)-3-BUTEN-2-ONE | 0.707739 | -0.06507 | 0.420974 | 0.132555 | 0.125591 |
| 5-[(4-BROMO-2-CHLOROPHENOXY)METHYL]FURAN-2-CARBALDEHYDE | 0.703644 | -0.41865 | 0.174486 | -0.28163 | -0.07651 |
| 5ALPHA-ANDROSTAN-17BETA-OL-3-ONE | 0.703547 | -0.10606 | 0.410103 | 0.214241 | 0.1553 |
| HYDROXYVALERENIC ACID | 0.702761 | -0.05477 | 0.400034 | 0.182468 | 0.070966 |
| ASTRAGALIN | 0.702262 | 0.018445 | -0.26637 | -0.25112 | 0.187103 |
| PICROSIDE III | 0.702079 | -0.00449 | -0.30693 | -0.22909 | 0.100002 |
| DIACETYL | 0.700032 | -0.04454 | -0.06772 | 0.512046 | 0.043208 |
| D-GALACTOSE | 0.698278 | -0.49541 | 0.103448 | -0.17208 | -0.11478 |
| DIANTHOSIDE | 0.697931 | 0.138211 | -0.10354 | -0.04964 | -0.19306 |
| DEHYDRO-L-(+)-ASCORBIC ACID DIMER | 0.693685 | -0.358 | -0.08579 | 0.161376 | 0.061732 |
| HOVETRICHOSIDE C | 0.691828 | -0.0576 | -0.44795 | 0.006935 | -0.24294 |
| DIMETHYL 4-HYDROXY-4-METHYL-2-(3-NITROPHENYL)-6-OXO-1,3-CYCLOHEXANEDICARBOXYLATE | 0.69149 | -0.49638 | 0.12491 | -0.16276 | -0.09702 |
| DILINOLENIN (9C,12C,15C) | 0.691041 | -0.16952 | -0.31111 | 0.245017 | 0.01117 |
| D-ARABINOSE | 0.690677 | -0.16933 | -0.18366 | -0.20514 | -0.04113 |
| PHENYLALANINE | 0.690401 | 0.271381 | 0.188253 | 0.30764 | -0.08812 |
| TRANS-4-(AMINOMETHYL)CYCLOHEXANECARBOXYLIC ACID | 0.689003 | -0.28376 | -0.02207 | -0.22146 | 0.198812 |
| 4-O-BETA-GALACTOPYRANOSYL-D-MANNOPYRANOSE | 0.688972 | -0.50578 | 0.125734 | -0.18144 | -0.12443 |
| 3-HYDROXY-3-METHYLGLUTARIC ACID | 0.687258 | -0.49197 | 0.132727 | -0.18214 | -0.11763 |
| FERULIC ACID METHYL ESTER | 0.684796 | 0.008043 | -0.1294 | -0.25407 | 0.083263 |
| 7,4'-DIMETHOXY-3-HYDROXYFLAVONE | 0.683457 | 0.381799 | 0.342308 | 0.025747 | 0.009352 |
| SUMARESINOLIC ACID | 0.681457 | -0.1896 | 0.347658 | -0.00187 | 0.182123 |
| EXO-NORBORNEOL | 0.67739 | -0.23317 | -0.01401 | -0.16448 | 0.235512 |
| NOTOGINSENOSIDE R2 | 0.677213 | -0.24698 | -0.11132 | -0.29717 | 0.090579 |
| N-(4-ETHYLBENZYL)-N-METHYLAMINE | 0.669525 | -0.029 | 0.371471 | 0.216507 | 0.138901 |
| KAEMPFEROL 3-O-RHAMNINOSIDE | 0.664251 | 0.324824 | -0.10046 | -0.43747 | 0.130739 |
| METHYL 2-(2,6-DIHYDROXYBENZOYL)-3-HYDROXY-5-METHYLBENZOATE | 0.65919 | -0.25126 | -0.15366 | -0.06918 | 0.164377 |
| (3BETA,6ALPHA,12BETA,20Z)-3,12-DIHYDROXYDAMMARA-20(22),24-DIEN-6-YL 2-O-(6-DEOXY-ALPHA-L-MANNOPYRANOSYL)-BETA-D-GLUCOPYRANOSIDE | 0.653833 | -0.24501 | -0.02522 | -0.32239 | 0.062471 |
| PRUNIN | 0.653144 | 0.346512 | 0.161059 | -0.39539 | -0.11497 |
| PHENYLACETYLASPARTIC ACID | 0.649474 | 0.372104 | 0.068654 | -0.40929 | -0.25251 |
| ORNITHINE | 0.64724 | -0.2581 | -0.25726 | -0.2164 | 0.032431 |
| 5-(6-METHYL-7-OXOOCTYL)FURAN-2(5H)-ONE | 0.644845 | -0.14932 | 0.412451 | 0.212547 | 0.188236 |
| CALYXANTHONE | 0.643277 | 0.359709 | 0.097574 | -0.20289 | -0.12987 |
| METHYL (9Z,14Z)-12,13,16-TRIHYDROXYOCTADECA-9,14-DIENOATE | 0.643255 | -0.13465 | 0.240734 | 0.359918 | 0.188684 |
| 2,4'-DIHYDROXYBENZOPHENONE | 0.641653 | -0.00194 | 0.014015 | -0.36804 | 0.023413 |
| GLYCITEIN | 0.64069 | 0.462101 | -0.27955 | -0.16345 | -0.34065 |
| 5,7-DIHYDROXY-2-(4-HYDROXYPHENYL)-6,8-BIS(3,4,5-TRIHYDROXYOXAN-2-YL)CHROMEN-4-ONE | 0.639797 | 0.240559 | 0.04128 | -0.38934 | -0.13063 |
| (3-NITROPHENYL)METHANAMINE | 0.633649 | -0.09081 | 0.188723 | 0.384868 | -0.07124 |
| PANTOTHENIC ACID | 0.633607 | 0.003713 | 0.2571 | -0.0602 | 0.2478 |
| TAXIFOLIN | 0.631131 | 0.07965 | 0.021937 | -0.42699 | 0.011162 |
| 2,6-DIHYDROXY-2-(4-HYDROXYBENZYL)-3-OXO-2,3-DIHYDRO-1-BENZOFURAN-4-YL 6-O-(6-DEOXY-ALPHA-L-MANNOPYRANOSYL)-BETA-D-GLUCOPYRANOSIDE | 0.630015 | 0.309439 | 0.043093 | -0.41936 | -0.13387 |
| KAEMPFEROL-3-O-ALPHA-L-ARABINOSIDE | 0.628091 | 0.297027 | -0.10415 | -0.40442 | 0.194804 |
| DATISCETIN | 0.62634 | 0.063564 | -0.26576 | -0.27031 | 0.088737 |
| CIS-11-METHYL-2-DODECENOIC ACID | 0.625559 | -0.20762 | 0.424769 | 0.098958 | 0.197438 |
| 3-(GALACTOSYLOXY)LINOLENIN | 0.622602 | -0.0315 | -0.17127 | 0.124418 | 0.119114 |
| NCGC00380867-01_C27H46O9_9,12,15-OCTADECATRIENOIC ACID, 3-(HEXOPYRANOSYLOXY)-2-HYDROXYPROPYL ESTER, (9Z,12Z,15Z)- | 0.619013 | -0.25081 | -0.49987 | 0.317549 | -0.01446 |
| 4',6-DIHYDROXY-3'-METHOXYAURONE | 0.618625 | 0.505859 | -0.26904 | -0.331 | -0.19918 |
| MELILOTOCARPAN B | 0.615169 | 0.540728 | -0.09891 | 0.059952 | -0.34881 |
| 2-AMINO-1-PHENYLETHANOL | 0.614996 | 0.292652 | 0.307558 | 0.263136 | 0.066933 |
| D-PYROGLUTAMIC ACID | 0.614525 | -0.50878 | 0.07277 | 0.392441 | -0.12161 |
| MACLURIN | 0.612215 | 0.075084 | 0.028647 | -0.40588 | 0.01754 |
| 1-(1-ADAMANTYLCARBONYL)-4-ETHYLPIPERAZINE | 0.607097 | -0.07271 | 0.452873 | 0.224592 | 0.135114 |
| NORWOGONIN | 0.603941 | 0.487086 | -0.08764 | 0.218862 | -0.25995 |
| ENGELETIN | 0.603844 | 0.080187 | 0.045452 | -0.40679 | 0.00114 |
| KAEMPFEROL 3-O-RUTINOSIDE | 0.603569 | -0.03161 | -0.31666 | -0.23493 | 0.07593 |
| 6,4'-DIMETHOXY-7-HYDROXYISOFLAVONE | 0.602051 | 0.300005 | 0.356425 | 0.06268 | 0.059067 |
| GNAPHALIIN | 0.60177 | 0.287698 | 0.140794 | -0.28966 | -0.40543 |
| DEHYDROASCORBIC ACID | 0.601708 | -0.40902 | -0.31891 | 0.088418 | 0.02311 |
| KAEMPFEROL-7-O-NEOHESPERIDOSIDE | 0.601552 | -0.05611 | -0.29897 | -0.24563 | 0.084186 |
| FORMONONETIN | 0.600651 | 0.472436 | -0.15542 | -0.3819 | -0.20451 |
| CIS-1,2,3,6-TETRAHYDROPHTHALIC ANHYDRIDE | 0.599727 | -0.05655 | -0.1798 | -0.17738 | -0.02186 |
| 2',4'-DIHYDROXYFLAVONE | 0.593712 | 0.445987 | -0.14256 | -0.47039 | -0.14359 |
| (-)-AROMADENDRIN | 0.592068 | 0.06741 | 0.030297 | -0.40624 | 0.023441 |
| GLUTAMINE | 0.590179 | -0.04496 | 0.113739 | 0.530959 | -0.12218 |
| NEGLETEIN | 0.589602 | 0.536388 | -0.10954 | -0.29035 | -0.27028 |
| (3BETA,9XI)-3-(BETA-D-GLUCOPYRANOSYLOXY)-14-HYDROXYCARD-20(22)-ENOLIDE | 0.588497 | -0.15856 | -0.47184 | 0.335711 | 0.014046 |
| CUNEATIN 7-GLUCOSIDE | 0.588392 | 0.327177 | 0.227942 | -0.19931 | -0.29829 |
| CITRULLINE | 0.582076 | -0.23625 | -0.1788 | -0.18249 | -0.09555 |
| PECTOLINARIN | 0.576548 | 0.491659 | 0.247944 | -0.21279 | -0.22509 |
| 4'-HYDROXY-3'-METHOXYFLAVONE | 0.576373 | 0.496328 | -0.16353 | -0.37301 | -0.18631 |
| ALOE-EMODIN | 0.576148 | 0.453057 | -0.14136 | -0.27002 | -0.23144 |
| CIRSIMARIN | 0.573661 | 0.502282 | 0.269467 | -0.19286 | -0.25775 |
| 3ALPHA-GALACTOBIOSE | 0.572342 | -0.4462 | 0.165295 | -0.28509 | -0.06393 |
| LOLIOLIDE | 0.567295 | 0.055824 | 0.288124 | 0.190603 | -0.0769 |
| 4-DEMETHYLSIMMONDSIN 2'-FERULATE | 0.566172 | -0.20157 | -0.18888 | -0.08139 | 0.148659 |
| KETOLOGANIC ACID | 0.563375 | -0.05958 | -0.19594 | -0.13821 | -0.01568 |
| (2E)-N-(4-ACETAMIDOBUTYL)-3-(4-HYDROXY-3-METHOXYPHENYL)PROP-2-ENAMIDE | 0.561895 | -0.19094 | -0.19184 | -0.0934 | 0.158711 |
| GINGERGLYCOLIPID A | 0.559819 | -0.29681 | -0.5189 | 0.317098 | -0.01985 |
| PROPAN-2-YL 6-O-(4-CARBOXY-3-HYDROXY-3-METHYLBUTANOYL)-BETA-D-GLUCOPYRANOSIDE | 0.552241 | -0.44371 | 0.228367 | -0.3539 | -0.13774 |
| ILE-LEU | 0.549483 | 0.342776 | 0.322532 | -0.18431 | 0.049751 |
| 1-MONOLINOLENIN | 0.546827 | -0.32916 | -0.45498 | 0.312689 | -0.08757 |
| N-ACETYLPHENYLALANINE | 0.544752 | 0.2608 | 0.421793 | -0.19716 | -0.09681 |
| APIGENIN | 0.543461 | 0.441278 | -0.14892 | -0.36081 | -0.16953 |
| GLUTAMATE | 0.541998 | -0.59147 | 0.109543 | 0.291515 | -0.08413 |
| N-ACETYL-D-MANNOSAMINE | 0.537933 | 0.011811 | -0.04823 | 0.182397 | 0.01984 |
| GLYCERATE | 0.537712 | -0.55656 | 0.128466 | 0.301046 | 0.018908 |
| TEXASIN | 0.533881 | 0.425792 | -0.26353 | -0.30311 | -0.22301 |
| N-ACETYL-L-GLUTAMINE | 0.533125 | -0.05684 | 0.113776 | 0.589244 | -0.17522 |
| LIQUIRITIGENIN | 0.530255 | 0.505847 | 0.057483 | -0.18953 | 0.065984 |
| GARBANZOL | 0.525675 | 0.073989 | -0.19348 | 0.166327 | 0.13349 |
| 4-((3R,4S)-4-HYDROXY-4-(4-HYDROXY-3-METHOXYBENZYL)-3-(HYDROXYMETHYL)TETRAHYDROFURAN-2-YL)-2-METHOXYPHENYL BETA-D-GLUCOPYRANOSIDE | 0.525184 | -0.14305 | -0.42643 | 0.29856 | 0.053999 |
| THREONIC ACID | 0.524614 | -0.46426 | 0.090802 | 0.207445 | -0.03524 |
| N-ACETYL-L-CARNOSINE | 0.519691 | 0.103742 | -0.25708 | 0.202637 | -0.17379 |
| ISORHAMNETIN-3-GLUCOSIDE | 0.516942 | 0.310022 | -0.32001 | -0.28855 | 0.200252 |
| ISORHOIFOLIN | 0.512428 | 0.107215 | -0.0472 | -0.3514 | 0.318788 |
| 6-HYDROXY-7-METHOXYCOUMARIN | 0.50951 | -0.23543 | 0.092982 | -0.18634 | 0.084451 |
| CROTONIC ACID | 0.508945 | 0.265541 | 0.017893 | 0.445567 | 0.247124 |
| SCOPOLETIN | 0.499475 | -0.2268 | 0.073625 | -0.19139 | 0.079879 |
| 2-METHYLIMIDAZOLE AZINE | 0.498258 | 0.019628 | -0.1956 | -0.2841 | 0.304585 |
| FLAVANOMAREIN | 0.497204 | 0.220658 | -0.02104 | 0.009571 | 0.211309 |
| (5E,7E)-3-ACETAMIDOUNDECA-5,7-DIENOIC ACID | 0.494018 | -0.34364 | -0.41513 | 0.430524 | -0.00576 |
| D-GLUCURONO-6,3-LACTONE | 0.491293 | -0.0426 | 0.003183 | 0.402338 | -0.04907 |
| ASPERLACTONE | 0.490827 | 0.251746 | -0.54428 | -0.27851 | 0.145028 |
| SUCCINIS ACID | 0.490023 | 0.110104 | 0.260033 | 0.591565 | -0.2519 |
| FRAXIDINGLUCOSIDE | 0.489741 | 0.267086 | 0.00971 | -0.32411 | -0.27831 |
| FRAXIDIN | 0.489111 | 0.295659 | -0.00318 | -0.30419 | -0.2654 |
| NALPHA-METHYL-L-LYSINE | 0.488933 | 0.121534 | 0.114894 | 0.058648 | 0.051395 |
| 3',4'-METHYLENEDIOXYOROBOL | 0.484828 | 0.41531 | -0.20214 | -0.28043 | 0.346932 |
| TRIFOLIRHIZIN | 0.483835 | 0.231286 | -0.54496 | -0.30753 | 0.147335 |
| OSTHENOL | 0.483411 | 0.239032 | -0.5361 | -0.28171 | 0.204776 |
| NCGC00385736-01_C42H64O16_ | 0.4823 | -0.09728 | -0.11804 | -0.04484 | 0.078672 |
| MMV687248 | 0.482017 | 0.174902 | -0.43611 | 0.469598 | -0.09516 |
| MAACKIAINE | 0.481668 | 0.607342 | -0.20832 | -0.07219 | 0.232118 |
| TECTORIDIN | 0.481606 | 0.591519 | -0.16016 | 0.262835 | -0.09038 |
| LEUCOSIDE | 0.480285 | -0.08585 | -0.45655 | -0.08715 | -0.21116 |
| DAMNACANTHAL | 0.479337 | 0.44386 | -0.23155 | -0.47461 | -0.21924 |
| GLUCARIC ACID | 0.47931 | 0.273452 | 0.256016 | -0.22138 | 0.078586 |
| 2-AMINOISOBUTYRIC ACID | 0.478284 | -0.54124 | 0.185389 | 0.382653 | -0.01803 |
| N-ALPHA-ACETYL-L-ARGININE | 0.477985 | 0.333755 | -0.20158 | 0.527196 | 0.03006 |
| DEOXYGUANOSINE | 0.477515 | -0.43265 | -0.06342 | 0.262679 | -0.24928 |
| NEPSILON-DIMETHYL-L-LYSINE | 0.477161 | 0.315656 | 0.228823 | 0.26674 | 0.364077 |
| ACETYLSALICYLSALICYLIC ACID | 0.475894 | 0.612929 | 0.012158 | -0.30325 | 0.114477 |
| 8-OXONONANOIC ACID | 0.475814 | -0.36556 | 0.127419 | -0.09647 | 0.262793 |
| ASP-LEU | 0.473196 | 0.530776 | 0.329896 | 0.379859 | 0.258212 |
| 4-(3,6-DIHYDROXY-2,3-BIS(HYDROXYMETHYL)-7-METHOXY-1,2,3,4-TETRAHYDRONAPHTHALEN-1-YL)-2-METHOXYPHENYL HEXOPYRANOSIDE | 0.472798 | -0.23247 | -0.26671 | 0.254311 | -0.05297 |
| ALA-LEU | 0.471126 | 0.237971 | 0.098061 | 0.483715 | 0.276772 |
| DAIDZEIN - 300 EV | 0.468514 | 0.5509 | -0.10707 | -0.41082 | -0.04074 |
| METHIONINE | 0.465151 | 0.113706 | -0.03216 | 0.642727 | 0.157901 |
| 7-HYDROXY-5-METHYL[1,2,4]TRIAZOLO[1,5-A]PYRIMIDIN-3-IUM-2-CARBOXYLATE | 0.46502 | 0.209954 | 0.118189 | -0.23899 | -0.15919 |
| 4-(2,6,6-TRIMETHYL-4-OXOCYCLOHEX-2-EN-1-YL)BUTAN-2-YL 6-O-(3,4,5-TRIHYDROXYBENZOYL)HEXOPYRANOSIDE | 0.459134 | 0.056629 | -0.44942 | 0.195078 | -0.31037 |
| 3-HYDROXY-2-((9Z,12Z)-OCTADECA-9,12-DIENOYLOXY)PROPYL 2-(TRIMETHYLAZANIUMYL)ETHYL PHOSPHATE | 0.458457 | 0.199684 | 0.184516 | 0.167642 | 0.428555 |
| MYO-INOSITOL, 1,2-ANHYDRO- | 0.458031 | 0.092427 | 0.093155 | 0.320581 | 0.020288 |
| 2-HYDROXYCINNAMIC ACID, PREDOMINANTLY TRANS | 0.457292 | -0.25167 | -0.33891 | -0.14993 | 0.497593 |
| COUMAROYL + C6H9O8 (ISOMER OF 843, 845, 846) | 0.455798 | -0.00328 | -0.01618 | -0.24028 | 0.025268 |
| 2-PHENYLETHYL 6-O-PENTOPYRANOSYLHEXOPYRANOSIDE | 0.452528 | 0.485524 | 0.056648 | -0.44793 | -0.21235 |
| HISPIDULIN 7-GLUCURONIDE | 0.452092 | 0.341095 | 0.174007 | -0.18191 | -0.09886 |
| KOPARIN | 0.450744 | 0.583807 | -0.17 | 0.363558 | -0.1122 |
| 3-(4-METHOXYPHENYL)-4-OXO-4H-CHROMEN-7-YL 6-O-(CARBOXYACETYL)-BETA-D-GLUCOPYRANOSIDE | 0.450495 | 0.5349 | -0.31833 | -0.43987 | 0.019794 |
| LEU-LEU | 0.448803 | 0.287728 | 0.09551 | -0.11622 | 0.096304 |
| CARVEOL | 0.443947 | 0.001351 | -0.46186 | 0.068382 | 0.016779 |
| 2-(METHYLTHIO)ACETOPHENONE | 0.443483 | -0.3479 | 0.172289 | -0.01239 | 0.274677 |
| CREATINE | 0.443164 | -0.47067 | -0.24464 | 0.076381 | -0.30576 |
| M-COUMARIC ACID | 0.443141 | -0.39741 | 0.313167 | -0.2876 | 0.294191 |
| LICOAGROSIDE B | 0.442953 | 0.013424 | -0.23629 | -0.23019 | -0.20821 |
| ILE-ILE | 0.441526 | 0.515239 | 0.251785 | -0.01621 | 0.217711 |
| 9(10)-EPOXY-12Z-OCTADECENOIC ACID | 0.439881 | -0.16166 | 0.049076 | -0.36042 | -0.11675 |
| D-GLUCURONIC ACID | 0.438874 | 0.147129 | -0.13741 | -0.00786 | -0.29953 |
| (2R,3S,4S,5R,6R)-2-[[(2S,3R,4R)-3,4-DIHYDROXY-4-(HYDROXYMETHYL)OXOLAN-2-YL]OXYMETHYL]-6-(2-PHENYLETHOXY)OXANE-3,4,5-TRIOL | 0.437152 | 0.445313 | 0.147075 | -0.43791 | -0.30271 |
| 2,6-DIAMINOPIMELIC ACID | 0.436368 | 0.307075 | 0.152883 | -0.11189 | 0.559788 |
| ROBUSTASIDE A | 0.432482 | -0.33057 | 0.185205 | -0.00368 | 0.256985 |
| RUTARENSIN | 0.431229 | -0.00886 | 0.226615 | -0.08925 | 0.102569 |
| DODECA-2(E),4(E)-DIENOIC ACID | 0.431083 | -0.26044 | -0.35088 | 0.320405 | -0.17898 |
| D-(+)-ARABITOL | 0.426923 | -0.26349 | -0.18638 | 0.450454 | -0.15928 |
| LYSINE | 0.42561 | 0.056078 | -0.36287 | 0.385238 | 0.066852 |
| THIAMINE | 0.425428 | -0.14091 | -0.0182 | 0.081175 | -0.00393 |
| LUCIDIN | 0.424965 | 0.555034 | 0.233978 | 0.217669 | -0.30188 |
| 7-HYDROXYCOUMARIN-3-CARBOXYLIC ACID | 0.424689 | 0.007528 | 0.409857 | -0.30365 | 0.178315 |
| GLYCYL-L-NORLEUCINE | 0.422563 | 0.287305 | 0.244734 | 0.420788 | 0.348373 |
| 1,2,3,3-TETRAMETHYLBICYCLO[221]HEPTAN-2-OL | 0.416779 | -0.35761 | 0.146366 | -0.078 | 0.244748 |
| 5,3'-DIHYDROXYFLAVONE | 0.413228 | 0.577062 | 0.021958 | 0.132743 | -0.03737 |
| LACTOBIONIC ACID | 0.412256 | 0.563488 | 0.145264 | 0.299014 | -0.15532 |
| 1,2-CYCLOHEXANEDIMETHANOL, 1,2-DIMETHANESULFONATE, (1R,2R)- | 0.409445 | -0.09434 | 0.02225 | -0.31373 | -0.13791 |
| MESACONIC ACID | 0.406313 | 0.0365 | -0.14633 | 0.592223 | -0.10902 |
| 3-(4-HYDROXY-3-METHOXYPHENYL)-4-OXO-4H-CHROMEN-7-YL HEXOPYRANOSIDE | 0.406276 | 0.443002 | 0.07299 | 0.124943 | 0.373694 |
| LINOLEOYLGLYCINE | 0.403313 | 0.047424 | 0.278591 | -0.14709 | 0.116819 |
| VAL-LEU | 0.40192 | 0.50951 | 0.343739 | 0.33214 | 0.287785 |
| 3-[5-(2-METHYLPROPYL)-3,6-DIOXOPIPERAZIN-2-YL]PROPANOIC ACID | 0.400463 | 0.562632 | 0.355201 | 0.450005 | 0.213886 |
| 3ALPHA-GALACTOBIOSE-CH3 | 0.399331 | -0.56199 | -0.23684 | 0.099116 | -0.0023 |
| 7-[(2E,5E)-7-HYDROXY-3,7-DIMETHYLOCTA-2,5-DIENOXY]CHROMEN-2-ONE | 0.397034 | 0.068354 | 0.415119 | -0.30408 | 0.187258 |
| 6'-MALONYLTRIFOLIRHIZIN | 0.394342 | 0.011816 | -0.33146 | 0.03863 | 0.295922 |
| 12,13-DIHOME | 0.394286 | -0.26707 | 0.607168 | -0.008 | -0.12238 |
| 6,2',3'-TRIHYDROXYFLAVONE | 0.393802 | 0.284564 | -0.50781 | -0.25777 | 0.121284 |
| LAETISARIC ACID | 0.392905 | 0.618322 | 0.033544 | 0.104866 | 0.111015 |
| ISOFRAXIDIN | 0.392748 | -0.00679 | -0.09378 | 0.12276 | -0.098 |
| 3-[6-[[(2R,3R,4R,5S,6S)-3,5-DIHYDROXY-6-METHYL-4-[(2S,3R,4R,5R,6S)-3,4,5-TRIHYDROXY-6-METHYLOXAN-2-YL]OXYOXAN-2-YL]OXYMETHYL]-3,4,5-TRIHYDROXYOXAN-2-YL]OXY-2-(3,4-DIHYDROXYPHENYL)-5,7-DIHYDROXYCHROMEN-4-ONE | 0.392056 | 0.086623 | -0.61676 | -0.22305 | 0.356666 |
| TRANS-ACONITIC ACID | 0.389032 | -0.01715 | -0.10723 | 0.579615 | -0.15752 |
| 1-AMINOCYCLOPENTANECARBOXYLIC ACID ETHYL ESTER | 0.387006 | -0.09189 | -0.07384 | 0.167627 | -0.18923 |
| 3,6,9,12-TETRAOXATETRACOSAN-1-OL | 0.386528 | 0.368022 | 0.072891 | -0.38152 | -0.23747 |
| ADENOSINE | 0.386122 | -0.13803 | 0.18573 | -0.01829 | 0.042269 |
| NEOLIQUIRITIN | 0.385998 | 0.612721 | 0.132747 | -0.30599 | 0.09488 |
| GLY-LEU | 0.383924 | 0.403111 | 0.179685 | 0.555433 | 0.210223 |
| GLU-LEU | 0.380589 | 0.518193 | 0.378079 | 0.40814 | 0.214425 |
| 1-METHYLADENOSINE | 0.379259 | 0.027824 | 0.105026 | -0.06478 | -0.36382 |
| PEONIDIN-3-O-BETA-D-GLUCOPYRANOSIDE | 0.378162 | 0.611965 | -0.11021 | 0.314245 | -0.12241 |
| PYRUVIC ACID | 0.378079 | 0.078988 | -0.16844 | 0.615816 | -0.07944 |
| PIPECOLIC ACID | 0.37483 | 0.34329 | 0.277193 | 0.287447 | 0.365355 |
| CITRIC ACID | 0.374825 | 0.044641 | -0.1824 | 0.584002 | -0.11667 |
| 6''-O-MALONYLGLYCITIN | 0.374078 | 0.618476 | -0.31361 | -0.11818 | 0.028922 |
| LEUCINE | 0.373002 | 0.481747 | -0.10551 | 0.57174 | -0.03895 |
| 1-(3,3-DIMETHYLBICYCLO[221]HEPT-2-YL)-1-PROPANOL | 0.370918 | 0.12914 | -0.10327 | 0.173796 | -0.43714 |
| SER-LEU | 0.370719 | 0.344477 | 0.294127 | 0.42046 | 0.338718 |
| FLAVONE BASE + 4O, O-MALONYLHEX | 0.370177 | 0.365125 | -0.05825 | -0.25954 | 0.12717 |
| 2,5-FURANDICARBOXYLIC ACID | 0.370005 | 0.056803 | -0.17047 | 0.602391 | -0.10518 |
| LINOELAIDIC ACID | 0.369132 | -0.10638 | 0.123397 | -0.31704 | -0.0973 |
| VALGANCICLOVIR | 0.368299 | -0.02899 | -0.41303 | 0.178242 | -0.00308 |
| SALICYLATE | 0.366363 | 0.431086 | 0.492462 | 0.059586 | -0.12884 |
| HYDROXYCAPROIC ACID | 0.365306 | 0.271259 | 0.191798 | -0.2067 | 0.271104 |
| 1-ACETYLPIPERIDINE-2-CARBOXYLIC ACID | 0.35821 | 0.36256 | 0.289598 | 0.310531 | 0.329098 |
| N-3-OXOHEXADEC-11Z-ENOYL-L-HOMOSERINE LACTONE | 0.356268 | -0.25399 | -0.05374 | -0.44397 | -0.00622 |
| CIS-10-HEPTADECENOIC ACID | 0.355485 | -0.2562 | -0.1063 | -0.14488 | 0.166811 |
| 3,6,2'-TRIHYDROXYFLAVONE | 0.353025 | 0.259996 | -0.49422 | -0.29649 | 0.02272 |
| 8-(3-OCTYL-2-OXIRANYL)OCTANOIC ACID | 0.348027 | -0.07519 | 0.0965 | -0.33333 | -0.09412 |
| HISTIDINE | 0.347163 | 0.099999 | -0.43881 | 0.366071 | -0.14805 |
| 1,6-BIS-O-(4-HYDROXYCINNAMOYL)GLUCOSE | 0.34687 | -0.60153 | 0.143302 | -0.18755 | 0.285609 |
| (1S,3R)-1-AMINOCYCLOPENTANE-1,3-DICARBOXYLIC ACID | 0.346806 | 0.131643 | 0.120908 | 0.207026 | 0.460999 |
| 10-HYDROXY-2-DECENOIC ACID | 0.340611 | -0.07736 | 0.132494 | 0.071863 | 0.504726 |
| 5-HYDROXY-2-(4-HYDROXYPHENYL)-7-((2-O-((2E)-3-(4-HYDROXYPHENYL)PROP-2-ENOYL)-BETA-D-GLUCOPYRANOSYL)OXY)-4-OXO-4H-CHROMEN-3-YL 2-O-(6-DEOXY-ALPHA-L-MANNOPYRANOSYL)-BETA-D-GLUCOPYRANOSIDE | 0.336516 | -0.44944 | -0.51758 | 0.198653 | -0.24672 |
| L-TYROSINE | 0.334056 | 0.58948 | -0.05178 | 0.56902 | 0.005007 |
| GLN-LEU | 0.331798 | 0.499719 | 0.294862 | 0.329196 | 0.425723 |
| GAMMA-DODECALACTONE | 0.318344 | 0.121099 | -0.10409 | 0.254487 | -0.20117 |
| ST024712 | 0.315719 | 0.453243 | 0.202827 | 0.2591 | 0.254141 |
| OCTYL ALPHA-KETOGLUTARATE | 0.314336 | 0.077578 | 0.218982 | 0.630324 | -0.11322 |
| LUTEOLIN | 0.31277 | 0.119988 | -0.53748 | -0.25546 | 0.162989 |
| 1,2,3,4-TETRAKIS(4-PYRIDYL)THIOPHENE | 0.306681 | -0.6025 | 0.063785 | -0.13795 | 0.273163 |
| 2,6-DIHYDROXYBENZOIC ACID | 0.306036 | 0.188171 | 0.393598 | -0.1055 | 0.051094 |
| OROXYLOSIDE | 0.305423 | 0.593919 | -0.3135 | -0.15533 | -0.00249 |
| SWEROSIDE | 0.303316 | 0.741229 | 0.272373 | -0.13699 | -0.15305 |
| GENISTIN | 0.30222 | 0.368908 | -0.1022 | 0.030962 | 0.290981 |
| 1-MONOLINOLEOYL-RAC-GLYCEROL | 0.302073 | -0.1536 | -0.01071 | -0.29646 | 0.006464 |
| 1-O-((2E,4E)-9-CARBOXY-8-HYDROXY-2,7-DIMETHYLNONA-2,4-DIENOYL)-BETA-D-GLUCOPYRANOSE | 0.299705 | 0.179269 | 0.455392 | -0.32001 | -0.29706 |
| D-ERYTHRONIC ACID GAMMA-LACTONE | 0.298658 | -0.29985 | 0.303184 | 0.206959 | 0.187986 |
| THR-LEU | 0.298346 | 0.135846 | 0.334673 | 0.428721 | 0.197877 |
| 1-O-((3ALPHA,5XI,9XI,16ALPHA,18XI)-16-HYDROXY-28-OXO-3-((4-O-BETA-D-XYLOPYRANOSYL-BETA-D-GLUCOPYRANURONOSYL)OXY)OLEAN-12-EN-28-YL)-BETA-D-GLUCOPYRANOSE | 0.298246 | -0.05675 | 0.264687 | 0.070658 | 0.303522 |
| KAEMPFEROL | 0.297906 | 0.173523 | -0.45358 | -0.18686 | 0.358347 |
| 3-(BENZOYLOXY)-2-HYDROXYPROPYL BETA-D-GLUCOPYRANOSIDURONIC ACID | 0.296074 | 0.070586 | 0.137631 | -0.29319 | 0.450623 |
| 10-UNDECYNOIC ACID | 0.292115 | -0.22692 | 0.160141 | 0.084415 | 0.138795 |
| QUERCETIN-3-O-BETA-D-XYLOPYRANOSYL (1-&gt;6)-BETA-D-GLUCOPYRANOSIDE | 0.29071 | -0.19079 | -0.75481 | 0.097429 | -0.1432 |
| PALMITAMIDE | 0.290591 | 0.119799 | 0.265325 | 0.183036 | 0.221442 |
| NEPETIN 7-GLUCOSIDE | 0.290497 | 0.370154 | 0.142797 | 0.063798 | -0.26683 |
| 3,4,5-TRIMETHACARB | 0.289918 | -0.01642 | 0.03755 | 0.275861 | 0.560066 |
| (4,7,7-TRIMETHYLBICYCLO[410]HEPT-4-EN-3-YL)METHANOL | 0.289418 | -0.49774 | 0.17832 | -0.00743 | 0.337298 |
| GUANOSINE | 0.288171 | 0.33884 | 0.010483 | 0.551403 | 0.113938 |
| OLEAMIDE | 0.287841 | -0.24364 | 0.103291 | -0.21451 | -0.07858 |
| ISOQUERCITIN | 0.286421 | -0.05222 | -0.79749 | -0.02789 | 0.181583 |
| ASN-LEU | 0.285892 | 0.562247 | 0.377714 | 0.396463 | 0.255262 |
| NARINGENIN | 0.285554 | 0.202526 | 0.015226 | -0.33595 | 0.198836 |
| 2-CYCLOHEXYL-N-(2-PHENYLETHYL)ACETAMIDE | 0.285179 | 0.183487 | 0.035657 | -0.35609 | -0.44427 |
| 2-HYDROXYHEXADECANOIC ACID | 0.285119 | 0.512218 | 0.143994 | 0.258986 | -0.10748 |
| 4-METHYLBENZENEMETHANOL | 0.28163 | 0.180878 | 0.043673 | -0.34394 | -0.44413 |
| 3'-DEOXYGUANOSINE | 0.280263 | -0.10792 | -0.03109 | 0.214437 | 0.190786 |
| (2S,3S,4S,5R,6R)-6-[[(3S,6AR,6BS,8AS,14BR)-4,4,6A,6B,11,11,14B-HEPTAMETHYL-8A-[(2S,3R,4S,5S,6R)-3,4,5-TRIHYDROXY-6-(HYDROXYMETHYL)OXAN-2-YL]OXYCARBONYL-1,2,3,4A,5,6,7,8,9,10,12,12A,14,14A-TETRADECAHYDROPICEN-3-YL]OXY]-3,4,5-TRIHYDROXYOXANE-2-CARBOXYLIC ACID | 0.279792 | -0.09604 | 0.111703 | -0.26267 | 0.592243 |
| 3-[3-(TRIFLUOROMETHYL)BENZYL]PIPERIDINE | 0.278945 | -0.38309 | 0.281263 | 0.021465 | 0.299171 |
| 5,7-DIHYDROXY-2-(4-HYDROXYPHENYL)-4-OXO-4H-CHROMEN-3-YL 6-DEOXY-ALPHA-L-MANNOPYRANOSYL-(1-&gt;2)-[6-DEOXY-ALPHA-L-MANNOPYRANOSYL-(1-&gt;6)]HEXOPYRANOSIDE | 0.278931 | -0.48429 | -0.18127 | 0.165771 | -0.33721 |
| 12(13)-EPOXY-9Z-OCTADECENOIC ACID | 0.275207 | -0.21971 | 0.499264 | -0.06688 | -0.06298 |
| ANTIBIOTIC NFAT 133 | 0.274488 | -0.09637 | 0.247932 | 0.039224 | 0.57238 |
| (3R)-3-HYDROXYDODECANOIC ACID | 0.274272 | -0.23426 | -0.45035 | 0.367543 | -0.30679 |
| (2S,3AS,7AS)-OCTAHYDRO-1H-INDOLE-2-CARBOXYLIC ACID | 0.27378 | -0.48791 | 0.243027 | -0.05706 | 0.29469 |
| ILE-ALA | 0.271661 | 0.322913 | 0.415151 | 0.17552 | -0.02757 |
| 3',4'-DIMETHOXY-7-HYDROXYISOFLAVONE | 0.268445 | 0.388927 | -0.13075 | -0.49754 | 0.074209 |
| ARGININE | 0.267395 | -0.19177 | -0.61481 | 0.279666 | -0.34688 |
| DODECANOIC ACID, 12-[[(TRICYCLO[33113,7]DEC-1-YLAMINO)CARBONYL]AMINO]- | 0.267141 | -0.25858 | -0.57191 | 0.338886 | -0.40035 |
| 5,7-DIHYDROXY-2-(4-HYDROXYPHENYL)-4-OXO-4H-CHROMEN-3-YL 6-DEOXYHEXOPYRANOSYL-(1-&gt;2)-[6-DEOXYHEXOPYRANOSYL-(1-&gt;6)]HEXOPYRANOSIDE | 0.266513 | -0.47334 | -0.32805 | 0.234518 | -0.39216 |
| EPOXYTRICARBALLYLIC ACID | 0.266289 | -0.0284 | 0.306555 | 0.122238 | 0.476041 |
| SIBIRICOSE A6 | 0.265313 | 0.012921 | -0.5832 | -0.11079 | 0.500956 |
| 3-((6-DEOXY-4-O-BETA-D-GLUCOPYRANOSYL-ALPHA-L-MANNOPYRANOSYL)OXY)-2-(3,4-DIHYDROXYPHENYL)-5-HYDROXY-4-OXO-4H-CHROMEN-7-YL HEXOPYRANOSIDE | 0.264138 | -0.05024 | -0.81224 | 0.035696 | 0.162744 |
| N-3-HYDROXYOCTANOYL-L-HOMOSERINE LACTONE | 0.262614 | -0.33576 | 0.282111 | -0.30703 | -0.0378 |
| SOPHORAFLAVONOLOSIDE | 0.260267 | -0.20138 | -0.77575 | 0.078249 | 0.084382 |
| 6-[[(3S,6AR,6BS,8AS,14BR)-4,4,6A,6B,11,11,14B-HEPTAMETHYL-8A-[3,4,5-TRIHYDROXY-6-(HYDROXYMETHYL)OXAN-2-YL]OXYCARBONYL-1,2,3,4A,5,6,7,8,9,10,12,12A,14,14A-TETRADECAHYDROPICEN-3-YL]OXY]-3,5-DIHYDROXY-4-[3,4,5-TRIHYDROXY-6-(HYDROXYMETHYL)OXAN-2-YL]OXYOXANE-2-CARBOXYLIC ACID | 0.259905 | -0.08976 | -0.32325 | 0.166285 | -0.21617 |
| (5XI,9XI,18XI)-6-HYDROXY-3-OXOLUP-20(29)-EN-28-OIC ACID | 0.259877 | -0.19905 | -0.03665 | -0.36721 | -0.14413 |
| BLOOD GROUP H DISACCHARIDE | 0.257398 | -0.07821 | 0.12747 | -0.06237 | -0.23753 |
| LEU-PRO | 0.255442 | 0.464012 | -0.00951 | 0.434349 | 0.350683 |
| DL-ARGININE | 0.25237 | -0.19608 | -0.6211 | 0.272574 | -0.31074 |
| LEU-GLU | 0.251107 | 0.468803 | 0.367144 | 0.301354 | 0.396411 |
| N-OLEOYLGLYCINE | 0.250842 | 0.25147 | 0.152019 | -0.09103 | 0.305578 |
| ISORHAMNETIN | 0.250221 | -0.38734 | -0.56361 | 0.191444 | -0.26919 |
| TETRADECANEDIOIC ACID | 0.250206 | -0.0302 | 0.18091 | -0.0324 | 0.588387 |
| KAEMPFEROL 3-O-(2,6-DI-O-ALPHA-L-RHAMNOPYRANOSYL)-BETA-D-GALACTOPYRANOSIDE | 0.247629 | -0.48776 | -0.33511 | 0.228783 | -0.39561 |
| 2,2-BIS(HYDROXYMETHYL)-3-QUINUCLIDINONE | 0.245533 | -0.4723 | 0.182173 | -0.04228 | 0.302877 |
| TRANS-TRAUMATIC ACID | 0.244882 | -0.09122 | -0.53044 | 0.431275 | -0.19241 |
| DODECANEDIOIC ACID | 0.242795 | -0.08184 | -0.23891 | 0.34666 | -0.31427 |
| OLEOYL ETHYLAMIDE | 0.241925 | 0.068498 | 0.189109 | -0.25576 | -0.07492 |
| DELTA-OCTALACTONE | 0.241221 | -0.48699 | 0.257738 | -0.05095 | 0.306356 |
| (+/-)-MEDICARPIN | 0.239755 | 0.390637 | -0.06179 | 0.184059 | 0.065413 |
| 2-AMINO-5-OXOHEXANOIC ACID | 0.238619 | 0.32968 | 0.083852 | -0.18165 | 0.473213 |
| 4-ANISALDEHYDE | 0.237412 | 0.382502 | -0.04722 | 0.207057 | 0.083366 |
| N-ACETYL-L-LEUCINE | 0.236397 | -0.46632 | 0.19011 | -0.07052 | 0.36734 |
| (2S,3S,4S,5R,6R)-6-[[(3S,4R,6AR,6BS,8AS,14BR)-4-(HYDROXYMETHYL)-4,6A,6B,11,11,14B-HEXAMETHYL-8A-[(2S,3R,4S,5S,6R)-3,4,5-TRIHYDROXY-6-(HYDROXYMETHYL)OXAN-2-YL]OXYCARBONYL-1,2,3,4A,5,6,7,8,9,10,12,12A,14,14A-TETRADECAHYDROPICEN-3-YL]OXY]-3,4,5-TRIHYDROXYOXANE-2-CARBOXYLIC ACID | 0.233966 | -0.15315 | 0.092 | -0.23552 | 0.579338 |
| NICOTINAMIDE | 0.231389 | 0.129868 | 0.499388 | 0.274271 | 0.140608 |
| 9-OXO-10E,12Z-OCTADECADIENOIC ACID | 0.230419 | -0.05256 | -0.09932 | -0.39782 | -0.29935 |
| SER-ILE | 0.229064 | 0.433597 | 0.331675 | 0.39079 | 0.333305 |
| 4-HYDROXY-4-[(E)-3-HYDROXYBUT-1-ENYL]-3,5,5-TRIMETHYLCYCLOHEX-2-EN-1-ONE | 0.228278 | 0.31575 | -0.15162 | -0.24026 | -0.33912 |
| (2Z)-2-(BETA-D-GLUCOPYRANOSYLOXY)-3-PHENYL-2-PROPENOIC ACID | 0.226089 | -0.27193 | -0.16299 | -0.0268 | 0.531353 |
| RUTIN | 0.220185 | -0.1439 | -0.78459 | 0.130147 | 0.100276 |
| SCLAREOLIDE | 0.218242 | 0.15129 | -0.23955 | 0.185909 | -0.37629 |
| 3-(2-HYDROXY-4-METHOXYPHENYL)-3,4-DIHYDRO-2H-CHROMEN-7-OL | 0.213005 | 0.495796 | 0.129786 | 0.618733 | -0.04702 |
| CHRYSOERIOL | 0.212581 | 0.418682 | -0.46442 | -0.28702 | 0.176005 |
| STEARAMIDE | 0.209306 | 0.354276 | 0.224359 | -0.33424 | 0.194883 |
| TYPHANEOSIDE | 0.20838 | -0.48533 | -0.44686 | 0.204078 | -0.31412 |
| 4-PENTEN-1-OL, 5-PHENYL-, (4E)- | 0.207785 | 0.222738 | -0.23744 | 0.155539 | -0.42381 |
| 5'-PRENYLPRATENSEIN | 0.205885 | 0.652888 | -0.24883 | -0.01972 | 0.00648 |
| (1R,5S)-9-METHYL-9-AZABICYCLO[331]NONAN-3-AMINE | 0.2022 | -0.49113 | 0.303559 | -0.03831 | 0.294599 |
| (4S)-4-AMINO-5-HYDROXYPENTANOIC ACID | 0.201518 | -0.03845 | 0.253476 | -0.24269 | 0.168855 |
| 4-[(2E)-3-(3,4-DIHYDROXYPHENYL)PROP-2-ENOYL]OXY-2,3-DIHYDROXY-2-METHYLBUTANOIC ACID | 0.201178 | 0.401023 | 0.047859 | 0.699386 | 0.098303 |
| 1-O-[(2ALPHA,3BETA,5XI,9XI)-3-(HEXOPYRANURONOSYLOXY)-2-HYDROXY-28-OXOOLEAN-12-EN-28-YL]HEXOPYRANOSE | 0.200195 | -0.02389 | 0.194049 | -0.14861 | 0.476683 |
| CEDROL | 0.199249 | -0.02091 | 0.163359 | -0.08614 | -0.13271 |
| FUMARIC ACID (NOT VALIDATED) | 0.198254 | 0.129963 | 0.151705 | 0.487228 | 0.135486 |
| 4-(HEXOPYRANOSYLOXY)-3-METHOXYBENZOIC ACID | 0.195602 | 0.273523 | 0.184715 | -0.02808 | 0.083518 |
| 3-(5-ACETYL-2-FURYL)-5-METHOXY-2-BENZOFURAN-1(3H)-ONE | 0.195262 | 0.293255 | 0.087721 | 0.12896 | -0.166 |
| DEOXYCARNITINE | 0.192892 | 0.279318 | 0.21012 | -0.38731 | -0.04877 |
| 9S-HYDROPEROXY-10E,12Z-OCTADECADIENOIC ACID | 0.192248 | 0.566054 | -0.08761 | 0.377898 | 0.129236 |
| EQUOL | 0.191503 | 0.468656 | 0.167513 | 0.61755 | -0.0764 |
| D-PSICOSE | 0.191433 | 0.069357 | 0.21092 | -0.11254 | -0.17909 |
| ZERUMBONE | 0.190112 | 0.157176 | -0.24574 | 0.18578 | -0.42725 |
| LUTEOLIN 7-METHYL ETHER | 0.188564 | 0.643122 | -0.08928 | -0.38468 | 0.240046 |
| [(2R,3S,4S,5R,6S)-3,4,5-TRIHYDROXY-6-[(2S,3R,4S,5S,6R)-3,4,5-TRIHYDROXY-6-(HYDROXYMETHYL)OXAN-2-YL]OXYOXAN-2-YL]METHYL 8-METHYLNONANOATE | 0.185021 | -0.20747 | 0.208615 | 0.084997 | -0.49323 |
| (E)-5-((1S,4R)-3,3-DIMETHYLBICYCLO[221]HEPTAN-2-YL)PENT-3-EN-2-ONE | 0.182678 | -0.01031 | 0.169417 | 0.043417 | 0.605518 |
| 1H-INDOLE-3-CARBOXAMIDE, 1-(5-CHLOROPENTYL)-N-1-NAPHTHALENYL- | 0.180149 | -0.04623 | 0.035758 | -0.12783 | -0.54517 |
| POLYGODIAL | 0.178303 | 0.164256 | -0.2391 | 0.203316 | -0.44148 |
| SKIMMIN | 0.177972 | 0.299369 | 0.284858 | -0.27359 | 0.444699 |
| 4-OXO-2-NONENAL | 0.174639 | 0.058671 | -0.01773 | 0.250186 | 0.633559 |
| 4-[(E)-5-HYDROXY-3-METHYLPENT-3-ENYL]-3,5,5-TRIMETHYLCYCLOHEX-2-EN-1-ONE | 0.174193 | -0.00365 | 0.157435 | -0.04716 | 0.62394 |
| 4-O-D-GLUCOPYRANOSYL-P-COUMARIC ACID | 0.173435 | -0.37046 | -0.29801 | 0.017588 | 0.384115 |
| 4(15)-SELINENE-11,12-DIOL | 0.172844 | 0.047341 | 0.181089 | -0.07518 | -0.15718 |
| VANILLIC ACID | 0.171553 | 0.395538 | 0.204167 | -0.02787 | 0.146791 |
| BENZOYLMALIC ACID | 0.168896 | 0.535416 | 0.068189 | -0.57875 | 0.174673 |
| 4H-1-BENZOPYRAN-4-ONE, 3-[[6-O-(6-DEOXY-ALPHA-L-MANNOPYRANOSYL)-BETA-D-GLUCOPYRANOSYL]OXY]-2-[4-(BETA-D-GLUCOPYRANOSYLOXY)PHENYL]-5,7- | 0.164826 | 0.030636 | -0.78733 | 0.017591 | 0.151087 |
| [(2R,3R,4S,5R,6S)-6-[(3S,4S,5S,6R)-4,5-DIHYDROXY-6-(HYDROXYMETHYL)OXAN-3-YL]OXY-3,4,5-TRIHYDROXYOXAN-2-YL]METHYL BENZOATE | 0.163374 | 0.152807 | 0.050229 | 0.426611 | -0.24952 |
| 1-PALMITOYL-2-LINOLEOYL-RAC-GLYCEROL | 0.162944 | -0.09517 | 0.047607 | 0.158876 | 0.253721 |
| PROLINE | 0.162142 | 0.255207 | -0.18916 | 0.684831 | -0.01648 |
| 5-(2-AMINOPROPYL)-2,3-DIHYDRO-1H-INDENE | 0.160409 | 0.26838 | -0.20275 | 0.156902 | -0.41588 |
| HEDEROSIDE F | 0.159603 | 0.04431 | 0.234415 | -0.03395 | -0.1401 |
| ISOKAEMPFERIDE | 0.159447 | 0.691276 | -0.14532 | -0.27806 | 7.46E-05 |
| 3-EPICOROSOLIC ACID | 0.158244 | 0.022417 | 0.187225 | -0.04758 | -0.13481 |
| N-FORMYLTRYPTOPHAN | 0.156548 | 0.159087 | -0.26282 | 0.510695 | 0.037533 |
| ALLANTOIC ACID | 0.156034 | -0.17318 | 0.318181 | 0.148195 | -0.11094 |
| VALERENIC ACID | 0.155516 | 0.031807 | 0.18066 | -0.05824 | -0.1388 |
| DIETHYL SUBERATE | 0.150049 | -0.42769 | 0.202466 | -0.06737 | 0.34778 |
| N-(2-CHLOROBENZYL)-2-(1H-INDOL-3-YL)ETHANAMINE | 0.14933 | 0.180425 | -0.26793 | 0.525221 | 0.037319 |
| DOCYNICASIDE A | 0.148631 | -0.12285 | -0.40006 | 0.35647 | -0.44274 |
| 2-(3,4-DIHYDROXYPHENYL)-3-[(2S,3R,4S,5S,6R)-4,5-DIHYDROXY-3-[(2R,3R,4R,5R,6S)-3,4,5-TRIHYDROXY-6-METHYLOXAN-2-YL]OXY-6-[[(2R,3R,4R,5R,6S)-3,4,5-TRIHYDROXY-6-METHYLOXAN-2-YL]OXYMETHYL]OXAN-2-YL]OXY-5,7-DIHYDROXYCHROMEN-4-ONE | 0.14842 | -0.07053 | -0.79112 | 0.089576 | 0.005281 |
| METHYL 3-(4-(BETA-D-GLUCOPYRANOSYLOXY)-2-(2-HYDROXYPROPAN-2-YL)-2,3-DIHYDRO-1-BENZOFURAN-5-YL)PROPANOATE | 0.144031 | -0.34196 | -0.24193 | -0.18071 | 0.135139 |
| SERINE | 0.14152 | 0.191291 | 0.081985 | 0.63434 | 0.034798 |
| 1-OLEOYL-2-PALMITOYL-RAC-GLYCEROL | 0.14001 | -0.03775 | 0.277235 | 0.418613 | 0.276319 |
| (10E,15Z)-9,12,13-TRIHYDROXYOCTADECA-10,15-DIENOIC ACID | 0.135816 | -0.23617 | 0.216397 | 0.40982 | -0.25417 |
| 3-CHLORO-5-METHYLBENZENE-1,2-DIOL | 0.134575 | 0.044298 | 0.462291 | -0.03573 | -0.16038 |
| (E)-3-[4-[(2S,3R,4S,5S,6R)-3,4,5-TRIHYDROXY-6-(HYDROXYMETHYL)OXAN-2-YL]OXYPHENYL]PROP-2-ENOIC ACID | 0.13229 | -0.31087 | -0.12352 | -0.00861 | 0.518747 |
| GLUTAMYLPHENYLALANINE | 0.130695 | 0.303652 | 0.159568 | -0.18206 | 0.352555 |
| 4-(2,6,6-TRIMETHYL-3-OXOCYCLOHEX-1-EN-1-YL)BUTAN-2-YL BETA-D-GLUCOPYRANOSIDE | 0.127488 | -0.04792 | -0.36364 | 0.470316 | -0.31777 |
| 2-PHENYLETHYL 2-O-BETA-D-XYLOPYRANOSYL-BETA-D-GLUCOPYRANOSIDE | 0.126474 | 0.307728 | 0.238226 | -0.45382 | -0.36436 |
| 2-PALMITOYL-RAC-GLYCEROL | 0.120834 | -0.08341 | 0.082581 | 0.287723 | 0.234161 |
| O6-MEG | 0.120403 | 0.023019 | 0.25881 | 0.027967 | -0.20767 |
| 1-CYCLOHEXYL-4-PIPERIDINAMINE | 0.117795 | -0.47213 | 0.330381 | 0.068695 | 0.241993 |
| 2-PHENYLETHYL 6-O-((2S,3R,4R)-3,4-DIHYDROXY-4-(HYDROXYMETHYL)TETRAHYDROFURAN-2-YL)-BETA-D-GLUCOPYRANOSIDE | 0.117322 | 0.301021 | 0.273843 | -0.42774 | -0.34382 |
| N-(4-BROMOPHENYL)ADAMANTAN-2-AMINE | 0.113081 | -0.5058 | 0.258826 | -0.06461 | 0.308854 |
| 3-[(1E,3E)-HEPTA-1,3-DIENYL]PENTANEDIOIC ACID | 0.112328 | 0.069212 | 0.393242 | -0.20304 | -0.28047 |
| NICOTINATE | 0.111041 | 0.334663 | 0.270214 | 0.617017 | -0.16894 |
| N-(4-CHLOROBENZOYL)TRYPTOPHAN | 0.110671 | 0.167106 | -0.2398 | 0.529226 | 0.040157 |
| ICARIDIN | 0.110485 | -0.41442 | 0.298927 | 0.110292 | 0.304569 |
| 5,7-DIHYDROXY-2-(4-HYDROXYPHENYL)-4-OXO-4H-CHROMEN-3-YL 6-DEOXY-2-O-(6-O-((2E)-3-(4-HYDROXYPHENYL)PROP-2-ENOYL)-BETA-D-GLUCOPYRANOSYL)-ALPHA-L-MANNOPYRANOSIDE | 0.110286 | -0.56324 | 0.284535 | -0.00797 | 0.294496 |
| (2R,3S,4S,5R,6S)-2-[[(2R,3R,4R)-3,4-DIHYDROXY-4-(HYDROXYMETHYL)OXOLAN-2-YL]OXYMETHYL]-6-PHENOXYOXANE-3,4,5-TRIOL | 0.110258 | 0.457672 | -0.22091 | 0.155766 | -0.22959 |
| 7-(HEXOPYRANOSYLOXY)-5-HYDROXY-2-(4-HYDROXYPHENYL)-4-OXO-4H-CHROMEN-3-YL 6-O-ACETYL-2-O-(6-DEOXY-ALPHA-L-MANNOPYRANOSYL)HEXOPYRANOSIDE | 0.108643 | 0.036485 | -0.11918 | 0.080176 | -0.14002 |
| 1,2-DIAMINO-2-METHYLPROPANE | 0.104679 | 0.384186 | -0.081 | 0.625261 | 0.105089 |
| FALLACINOL | 0.104418 | 0.24409 | -0.14345 | 0.280045 | -0.33853 |
| HYDROXYISOCAPROIC ACID | 0.104296 | 0.191865 | 0.170999 | -0.28944 | 0.35383 |
| 1,2-DIOLEOYL-SN-GLYCERO-3-PHOSPHATIDYLCHOLINE | 0.102596 | 0.299825 | -0.07074 | -0.37402 | 0.100766 |
| N-ACETYL-L-TYROSINE ETHYL ESTER | 0.100614 | 0.505837 | 0.178094 | 0.257741 | -0.20684 |
| D-TYROSINE TERT-BUTYL ESTER | 0.09738 | -0.42629 | 0.231597 | -0.05117 | 0.299917 |
| 4-ISOPROPYL-3,4,5,6,7,8-HEXAHYDRO-2(1H)-QUINAZOLINETHIONE | 0.092812 | -0.49151 | 0.284486 | -0.08742 | 0.325579 |
| (Z)-3-METHYL-5-(1,2,5,5-TETRAMETHYL-7-OXO-3,4,4A,6-TETRAHYDRO-2H-NAPHTHALEN-1-YL)PENT-2-ENOIC ACID | 0.09281 | -0.12977 | 0.620268 | 0.231687 | -0.4983 |
| CAULOPHYLLOGENIN | 0.09248 | 0.039212 | -0.02032 | -0.17709 | -0.10017 |
| N-ACETYLTRYPTOPHAN | 0.087161 | -0.10102 | 0.337069 | -0.0522 | -0.16297 |
| MALATE | 0.086835 | 0.138257 | 0.097683 | 0.43983 | 0.144458 |
| MALIOXAMYCIN | 0.083689 | -0.2624 | 0.385062 | 0.152399 | -0.28586 |
| TILIROSIDE | 0.081133 | -0.51205 | 0.151886 | -0.01744 | 0.298055 |
| TRYPTOPHAN | 0.076183 | 0.141678 | -0.25703 | 0.496207 | 0.025429 |
| (3E)-4-(2,6,6-TRIMETHYL-4-OXOCYCLOHEX-2-EN-1-YL)BUT-3-EN-2-YL 2-O-((2S,3R,4R)-3,4-DIHYDROXY-4-(HYDROXYMETHYL)TETRAHYDROFURAN-2-YL)-BETA-D-GLUCOPYRANOSIDE | 0.074963 | -0.11108 | -0.3519 | 0.320575 | 0.333513 |
| ADENINE | 0.069714 | 0.377901 | 0.319249 | 0.482761 | -0.06698 |
| 1-(P-TOLYL)CYCLOPROPANECARBONITRILE | 0.06957 | 0.338429 | 0.027322 | 0.241599 | -0.38965 |
| 4-HYDROXYNONENAL ALKYNE | 0.064982 | -0.52927 | 0.223403 | 0.174953 | 0.107779 |
| 4-(BETA-D-GLUCOPYRANOSYLOXY)BENZYL 3-(BETA-D-GLUCOPYRANOSYLOXY)-2-(((2Z)-3-(4-HYDROXYPHENYL)PROP-2-ENOYL)OXY)-3-METHYLBUTANOATE | 0.063864 | -0.3075 | -0.10327 | 0.414529 | 0.230876 |
| 4'-METHOXYFLAVANONE | 0.055946 | 0.722154 | 0.34649 | 0.271271 | 0.19927 |
| ALEURITIC ACID | 0.047964 | -0.05996 | 0.41325 | 0.024224 | 0.065455 |
| BIOCHANIN A | 0.046913 | 0.704577 | -0.10131 | 0.101108 | -0.24223 |
| INDIGOTIN | 0.04639 | -0.32791 | 0.141784 | 0.061585 | -0.26355 |
| 2-HYDROXY-6-AMINOPURINE | 0.040832 | -0.17582 | 0.047689 | 0.362787 | 0.011755 |
| 1-O-((3BETA,5XI,9XI,18XI)-3-(BETA-D-GLUCOPYRANURONOSYLOXY)-23-HYDROXY-28-OXOOLEAN-12-EN-28-YL)-BETA-D-GLUCOPYRANOSE | 0.040762 | -0.03813 | 0.217783 | -0.00915 | 0.409063 |
| PYRIDINE | 0.032743 | -0.06615 | 0.042039 | -0.04381 | 0.158659 |
| 2-(4-AMINO-1-PIPERIDINYL)CYCLOPENTANOL | 0.028467 | -0.07223 | 0.120548 | 0.263647 | 0.240846 |
| DAPHNIN | 0.027757 | 0.425211 | 0.021098 | -0.20876 | 0.39832 |
| 3,4-DIHYDROXYCINNAMIC ACID (L-ALANINE METHYL ESTER) AMIDE | 0.027662 | 0.289786 | -0.15626 | -0.05649 | 0.636589 |
| 2,3-DIHYDROXY-2-METHYLBUTANOIC ACID | 0.027021 | -0.18315 | -0.40165 | 0.200075 | 0.197989 |
| PRUNETINOSIDE | 0.026781 | 0.525197 | -0.14086 | -0.19415 | 0.163562 |
| 2-GLUCOSYLOXY-4-METHOXYCINNAMIC ACID | 0.026065 | 0.463385 | 0.039801 | 0.573552 | 0.334005 |
| 2-[[3,4-DIHYDROXY-4-(HYDROXYMETHYL)OXOLAN-2-YL]OXYMETHYL]-6-[(2-HYDROXY-4,7,7-TRIMETHYL-5-BICYCLO[221]HEPTANYL)OXY]OXANE-3,4,5-TRIOL | 0.025507 | -0.24842 | 0.213342 | 0.059229 | -0.52197 |
| TUBERONIC ACID GLUCOSIDE | 0.020347 | 0.042133 | 0.344916 | -0.22897 | -0.36908 |
| SIBIRICOSE A3 | 0.019703 | 0.185233 | -0.54342 | 0.055824 | 0.536772 |
| CEDEODARIN | 0.018727 | 0.221609 | -0.09767 | 0.183875 | -0.35629 |
| BETA-CAROTENE | 0.010766 | -0.00826 | 0.298254 | -0.12678 | -0.14315 |
| DIOSMETIN | 0.009667 | 0.159522 | -0.57525 | 0.156318 | -0.09837 |
| (R)-AMINOCARNITINE | 0.005905 | -0.41357 | 0.295784 | 0.13349 | -0.07555 |
| 3-(DIMETHYLAMINO)PROPIOPHENONE | 0.000418 | -0.21455 | 0.326987 | 0.101797 | -0.42272 |
| L-ISOLEUCINE | -0.01495 | 0.358279 | -0.07074 | 0.348001 | -0.12425 |
| 2-METHYLBUT-3-EN-2-YL 6-O-((2R,3R,4R)-3,4-DIHYDROXY-4-(HYDROXYMETHYL)TETRAHYDROFURAN-2-YL)-BETA-D-GLUCOPYRANOSIDE | -0.02484 | 0.003222 | -0.55187 | -0.05295 | -0.16543 |
| N-(4-METHYLPHENYL)-2-(1-METHYL-3-PHENYLTHIOINDOL-2-YL)ACETAMIDE | -0.04088 | -0.06852 | -0.2573 | 0.203433 | 0.214641 |
| AMINOADIPATE | -0.04902 | 0.130078 | 0.089853 | 0.071463 | 0.342264 |
| ESCULIN | -0.05503 | 0.300128 | -0.20008 | -0.08726 | 0.641293 |
| (4Z)-4-(2,6,6-TRIMETHYL-4-OXOCYCLOHEX-2-EN-1-YLIDENE)BUTAN-2-YL 6-O-BETA-D-XYLOPYRANOSYL-BETA-D-GLUCOPYRANOSIDE | -0.06545 | -0.17033 | -0.41615 | 0.39519 | 0.318721 |
| (+/-)7-EPI-JASMONIC ACID | -0.06701 | -0.31146 | 0.402158 | -0.01348 | -0.34847 |
| ETHYL 4-HYDROXYQUINOLINE-2-CARBOXYLATE | -0.07692 | -0.06374 | -0.37184 | 0.192759 | 0.107268 |
| 4-HYDROXY-6-[2-(2-METHYL-1,2,4A,5,6,7,8,8A-OCTAHYDRONAPHTHALEN-1-YL)ETHYL]OXAN-2-ONE | -0.0775 | -0.08024 | 0.671477 | 0.180011 | -0.44287 |
| 2,3-DINOR-11BETA-PROSTAGLANDIN F2ALPHA | -0.0785 | -0.05621 | 0.698723 | 0.148632 | -0.18544 |
| PLATANIONOSIDE C | -0.0792 | 0.052419 | 0.01129 | 0.476084 | 0.196158 |
| 2(5H)-FURANONE, 5-(7-HYDROXY-6-METHYLOCTYL)- | -0.08066 | 0.040573 | -0.38996 | 0.307165 | -0.44441 |
| (E)-ALPHA-IONONE | -0.08079 | 0.056616 | -0.1011 | 0.564013 | 0.089058 |
| 18BETA-GLYCYRRHETINIC ACID | -0.09357 | 0.314601 | 0.117019 | -0.15593 | 0.131188 |
| CHROMOMORIC ACID B | -0.09478 | 0.199175 | 0.655368 | 0.175696 | -0.01589 |
| COLUBRINIC ACID | -0.09501 | 0.336322 | 0.140311 | -0.16388 | 0.166047 |
| 3-(2-(4-HYDROXY-3-METHOXYPHENYL)-3-(HYDROXYMETHYL)-7-METHOXY-2,3-DIHYDRO-1-BENZOFURAN-5-YL)PROPYL BETA-D-GLUCOPYRANOSIDE | -0.09581 | -0.38295 | 0.234624 | 0.137529 | 0.072779 |
| SULFO JASMONATE | -0.09691 | 0.118921 | 0.342405 | -0.37788 | -0.34755 |
| 5-[(1E,3E)-HEPTA-1,3-DIENYL]CYCLOHEXANE-1,2,3-TRIOL | -0.09694 | 0.051625 | -0.37757 | 0.319604 | -0.43386 |
| N-(AMINOTHIOXOMETHYL)-5-OXO-1-PROPYL-2-PYRROLIDINEACETAMIDE | -0.11551 | -0.23022 | 0.324155 | 0.062197 | -0.43019 |
| (12Z,15Z)-9,10,11-TRIHYDROXYOCTADECA-12,15-DIENOIC ACID | -0.11595 | -0.03558 | 0.35608 | 0.340676 | -0.2168 |
| VAL-THR | -0.11817 | 0.520559 | 0.16318 | 0.204771 | 0.286048 |
| 4-(2-CYCLOHEXYLETHOXY)ANILINE | -0.12215 | -0.23549 | 0.260187 | 0.18 | -0.33802 |
| 4-HYDROXY-3,7-QUINOLINEDICARBOXYLIC ACID | -0.12335 | -0.0805 | -0.40781 | 0.145615 | 0.074332 |
| 3-HYDROXYDODECANOIC ACID | -0.13278 | 0.093308 | -0.35968 | 0.275334 | -0.45677 |
| 1-O-(3-((6-O-(6-DEOXYHEXOPYRANOSYL)HEXOPYRANOSYL)OXY)-23-HYDROXY-28-OXOOLEAN-12-EN-28-YL)HEXOPYRANOSE | -0.13422 | 0.101298 | 0.441421 | -0.46035 | 0.009325 |
| ALA-PRO | -0.15185 | -0.15058 | 0.175612 | 0.150926 | 0.127586 |
| 3-METHYLBUTYL 6-O-ALPHA-L-ARABINOPYRANOSYL-BETA-D-GLUCOPYRANOSIDE | -0.15781 | -0.13579 | -0.17559 | -0.12893 | -0.49689 |
| 1-OLEOYL-2-PALMITOYL-SN-GLYCERO-3-PHOSPHOCHOLINE | -0.16171 | 0.170152 | 0.024992 | -0.01652 | -0.22491 |
| (2S,3S,4S,5R,6R)-6-[[(3S,6AR,6BS,8AS,14BR)-8A-CARBOXY-4-(HYDROXYMETHYL)-4,6A,6B,11,11,14B-HEXAMETHYL-1,2,3,4A,5,6,7,8,9,10,12,12A,14,14A-TETRADECAHYDROPICEN-3-YL]OXY]-3,4,5-TRIHYDROXYOXANE-2-CARBOXYLIC ACID | -0.16181 | -0.02867 | 0.436772 | 0.06943 | 0.472319 |
| FORMONONETIN-7-O-GLUCOSIDE | -0.16304 | 0.393657 | -0.20386 | -0.24554 | 0.158019 |
| 1,3,4,6-TETRA-O-ACETYL-BETA-D-MANNOPYRANOSE | -0.16759 | 0.227593 | -0.48153 | 0.148319 | 0.010034 |
| 1,6-ANHYDRO-2,3-O-ISOPROPYLIDENE-BETA-D-MANNOPYRANOSE | -0.17158 | -0.04974 | -0.06178 | 0.189145 | 0.413792 |
| 2-{2-[2-(ACRYLOYLOXY)-1-METHYLETHOXY]-1-METHYLETHOXY}-1-METHYLETHYL ACRYLATE | -0.17578 | -0.19073 | 0.004142 | -0.30502 | -0.06965 |
| 2-(3,4-DIHYDROXYPHENYL)-5,7-DIHYDROXY-4-OXO-4H-CHROMEN-3-YL 4-O-HEXOPYRANOSYLHEXOPYRANOSIDE | -0.17939 | 0.039963 | -0.73801 | 0.100845 | 0.352427 |
| 1-O-((2BETA,3BETA,5XI,9XI,18XI)-3-(BETA-D-GLUCOPYRANURONOSYLOXY)-2,23-DIHYDROXY-28-OXOOLEAN-12-EN-28-YL)-BETA-D-GLUCOPYRANOSE | -0.20521 | -0.10873 | -0.19644 | -0.26183 | -0.12372 |
| METHYL 3,8-DIHYDROXY-6-METHYL-9-OXOXANTHENE-1-CARBOXYLATE | -0.21067 | 0.3063 | -0.18578 | -0.24574 | -0.0141 |
| QUERCETIN 3,4'-DIGLUCOSIDE | -0.21163 | 0.081894 | -0.68217 | 0.088707 | 0.403343 |
| BENZYL 6-O-(4-CARBOXY-3-HYDROXY-3-METHYLBUTANOYL)HEXOPYRANOSIDE | -0.21185 | 0.24073 | 0.178243 | 0.322217 | 0.046646 |
| 1,2-DILINOLEOYL-SN-GLYCERO-3-PHOSPHOCHOLINE | -0.21268 | 0.035426 | -0.26607 | -0.12725 | 0.026373 |
| 1,2-DIPALMITOYL-SN-GLYCERO-O-ETHYL-3-PHOSPHATIDYLCHOLINE CATION | -0.2158 | 0.141307 | -0.19629 | -0.15431 | -0.06706 |
| ONONIN | -0.21718 | 0.329076 | -0.21068 | -0.27507 | 0.128875 |
| N-ACETYLPROLINE | -0.22055 | 0.326827 | 0.216659 | 0.327815 | 0.149509 |
| 2-OLEOYL-1-PALMITOYL-SN-GLYCERO-3-PHOSPHOCHOLINE | -0.22177 | 0.032981 | -0.27425 | -0.13924 | 0.026722 |
| PHENANTHRO[3,2-B]FURAN-9(1H)-ONE, 2,3,4,4A,5,6,6A,7,10A,11,11A,11B-DODECAHYDRO-6,10A-DIHYDROXY-4-(HYDROXYMETHYL)-4,7,11B-TRIMETHYL- | -0.22701 | 0.04754 | 0.369573 | 0.288758 | -0.19432 |
| QUINOLIN-3-OL | -0.22883 | 0.40627 | 0.287676 | 0.307433 | -0.06799 |
| 5-(GALACTOSYLHYDROXY)-L-LYSINE | -0.23226 | -0.26958 | 0.313034 | 0.11893 | -0.26517 |
| (2R,3S,4S,5S,6R)-2-[[7-HYDROXY-4-[(Z)-5-HYDROXY-3-METHYLPENT-3-ENYL]-4A,8,8-TRIMETHYL-3-METHYLIDENE-2,4,5,6,7,8A-HEXAHYDRO-1H-NAPHTHALEN-2-YL]OXY]-6-(HYDROXYMETHYL)OXANE-3,4,5-TRIOL | -0.23278 | 0.015767 | -0.42345 | 0.333657 | 0.394121 |
| 1-STEAROYL-2-ARACHIDONYL-SN-GLYCERO-3-PHOSPHOCHOLINE | -0.23283 | 0.03272 | -0.2673 | -0.13894 | 0.025382 |
| 1-PALMITOYL-SN-GLYCERO-3-PHOSPHOCHOLINE | -0.23672 | 0.067932 | -0.27057 | -0.15751 | 0.004414 |
| PALMITOYL SPHINGOMYELIN | -0.24788 | 0.042214 | -0.26127 | -0.16085 | 0.019464 |
| N,N,N-TRIMETHYL-5-[(TRICYCLO[3311(3,7)]DEC-1-YLMETHYL)AMINO]-1-PENTANAMINIUM CATION | -0.25002 | 0.235441 | 0.291314 | 0.181061 | -0.17569 |
| 13S-HYDROPEROXY-9Z,11E-OCTADECADIENOIC ACID | -0.25263 | 0.054317 | 0.463953 | -0.02003 | -0.36251 |
| 1-PALMITOYL-2-LINOLEOYL-SN-GLYCERO-3-PHOSPHOCHOLINE | -0.25284 | 0.02994 | -0.26805 | -0.16052 | 0.032145 |
| 4-ACETYLOXY-8-(3-OXO-2-PENT-2-ENYLCYCLOPENTEN-1-YL)OCTANOIC ACID | -0.2548 | -0.02689 | 0.522146 | 0.14021 | -0.40441 |
| (Z)-3-HEXENYLVICIANOSIDE | -0.27872 | -0.10547 | 0.314131 | -0.10195 | -0.43711 |
| MARITIMEIN | -0.27953 | 0.267533 | -0.18414 | -0.20104 | -0.09393 |
| 4',7-DIMETHOXYISOFLAVONE | -0.28006 | 0.039763 | 0.082357 | -0.25865 | -0.10299 |
| OXOPROLINE | -0.2809 | 0.021938 | 0.168593 | 0.259156 | 0.334965 |
| ASTERRIC ACID | -0.28617 | 0.279761 | 0.193619 | -0.24355 | -0.26606 |
| PALMITOYLEICOSAPENTAENOYL PHOSPHATIDYLCHOLINE | -0.28738 | -0.00093 | -0.25509 | -0.07532 | 0.114468 |
| SORBITOL | -0.30929 | 0.49614 | 0.169586 | -0.35662 | -0.05755 |
| (9Z)-5,8,11-TRIHYDROXYOCTADEC-9-ENOIC ACID | -0.31416 | 0.055222 | 0.441112 | 0.145628 | -0.28378 |
| PRO-LEU | -0.31449 | 0.314725 | 0.062557 | 0.016705 | -0.0339 |
| OSMANTHUSIDE H | -0.31767 | 0.098422 | -0.28717 | -0.27965 | 0.061826 |
| DIKEGULAC ACID | -0.31895 | 0.045416 | -0.3268 | 0.034923 | 0.297368 |
| MADECASSIC ACID | -0.3247 | 0.270067 | 0.021023 | 0.112903 | 0.025736 |
| DIETHYLENE GLYCOL MONOBUTYL ETHER | -0.32924 | 0.05893 | 0.272272 | 0.009867 | -0.04228 |
| ECTOINE | -0.3303 | -0.14816 | 0.270494 | 0.034602 | 0.132101 |
| (12Z,15Z)-7,9,10-TRIHYDROXYOCTADECA-12,15-DIENOIC ACID | -0.33137 | 0.048185 | 0.598494 | 0.10722 | -0.37386 |
| BETA-D-GLUCOPYRANOSIDE, PHENYLMETHYL 6-O-[(2R,3R,4R)-TETRAHYDRO-3,4-DIHYDROXY-4-(HYDROXYMETHYL)-2-FURANYL]- | -0.33675 | 0.449495 | 0.168451 | -0.25668 | -0.31723 |
| CREATININE | -0.34459 | -0.17811 | 0.170061 | -0.00227 | 0.184803 |
| THREONINE | -0.34497 | 0.274765 | 0.172858 | 0.628984 | 0.055549 |
| CITRAMALIC ACID | -0.3508 | -0.36085 | 0.141916 | 0.083732 | -0.11641 |
| TETRAHYDRODICRANENONE B | -0.35094 | 0.154566 | 0.466474 | 0.176962 | -0.3167 |
| 1,6-ANHYDRO-BETA-D-GLUCOSE | -0.3613 | 0.228036 | -0.30996 | 0.142638 | -0.0365 |
| BENZYL 6-O-(4-CARBOXY-3-HYDROXY-3-METHYLBUTANOYL)-BETA-D-GLUCOPYRANOSIDE | -0.38156 | 0.269094 | 0.256411 | 0.349662 | 0.117707 |
| ALA-ALA | -0.38798 | -0.07993 | 0.147293 | 0.041624 | 0.187131 |
| 5-[2-(FURAN-3-YL)ETHYL]-8-HYDROXY-5,6,8A-TRIMETHYL-3,4,4A,6,7,8-HEXAHYDRONAPHTHALENE-1-CARBOXYLIC ACID | -0.38851 | 0.008637 | 0.333559 | 0.30709 | -0.11761 |
| 7-[2-(FURAN-3-YL)ETHYL]-5-HYDROXY-7,8-DIMETHYL-5,6,6A,8,9,10-HEXAHYDRO-1H-BENZO[D][2]BENZOFURAN-3-ONE | -0.38851 | 0.107184 | 0.288305 | 0.28335 | -0.46897 |
| 6-HYDROXYNICOTINATE | -0.38988 | -0.00293 | 0.338412 | -0.07444 | 0.073208 |
| 3-BUTEN-2-ONE, 4-[4-(BETA-D-GLUCOPYRANOSYLOXY)-2-HYDROXY-2,6,6-TRIMETHYLCYCLOHEXYLIDENE]- | -0.39406 | 0.176817 | -0.41695 | 0.517454 | 0.029726 |
| PROSTAGLANDIN A1 ETHYL ESTER | -0.39593 | 0.379032 | 0.513177 | -0.21633 | -0.09501 |
| SYRINGALDEHYDE | -0.40447 | 0.107068 | 0.495648 | 0.187737 | -0.4022 |
| CINNABARINIC ACID | -0.40587 | -0.13129 | 0.143238 | 0.077656 | 0.220115 |
| BENZOIC ACID + 2O, O-PEN | -0.40949 | 0.440707 | 0.066191 | 0.409429 | 0.234006 |
| 5'-O-ACETYL-(R)-LISOFYLLINE | -0.41286 | 0.121008 | -0.19388 | 0.409876 | -0.03435 |
| 3-CYCLOPENTENE-1-OCTANOIC ACID, 2-(3-HYDROXY-1-PENTEN-1-YL)-5-OXO- | -0.42558 | 0.195593 | 0.30491 | 0.125361 | -0.29638 |
| 4-(HYDROXYMETHYL)-4,6A,9,12B-TETRAMETHYL-4A,6,6A,12,12A,12B-HEXAHYDRO-4H,9H-BENZO[F]PYRANO[2,3-B]CHROMENE-1,11(5H,10H)-DIONE | -0.43398 | -0.01983 | -0.30733 | 0.417274 | 0.134391 |
| METHIONINESULFOXIDE | -0.43499 | 0.38372 | 0.155475 | -0.05021 | 0.042656 |
| NICOTINIC ACID MONONUCLEOTIDE | -0.43885 | -0.12356 | 0.350599 | -0.0542 | 0.325889 |
| N-ACETYL-DL-VALINE | -0.4412 | 0.082418 | -0.06878 | 0.140616 | 0.319701 |
| (3R)-4,4-DIMETHYL-3-[(2S,3R,4S,5S,6R)-3,4,5-TRIHYDROXY-6-(HYDROXYMETHYL)OXAN-2-YL]OXYOXOLAN-2-ONE | -0.44397 | 0.085754 | -0.06296 | -0.37443 | 0.150042 |
| 8R-HYDROXY-4Z,6E,10Z-HEXADECATRIENOIC ACID | -0.44453 | 0.059614 | 0.413578 | 0.007985 | -0.30391 |
| 2-PHENYL-5H,6H-[1,2,4]TRIAZOLO[1,5-C]QUINAZOLIN-5-ONE | -0.44628 | -0.10606 | 0.124465 | 0.05464 | 0.217322 |
| NAPHTHALENE-2-SULFONIC ACID | -0.44653 | 0.19367 | -0.00316 | -0.01713 | 0.014572 |
| 2,5-DIMETHYL-4-PYRIMIDINAMINE | -0.44972 | -0.07906 | 0.036723 | 0.099907 | 0.278719 |
| GERMACRONE | -0.45233 | 0.050195 | 0.361401 | 0.107094 | -0.3283 |
| 9-HOTRE | -0.45633 | 0.178087 | 0.408898 | 0.17823 | -0.37028 |
| 1,4-D-XYLOBIOSE | -0.46341 | 0.229893 | -0.28707 | 0.093841 | 0.407802 |
| LOXISTATIN ACID | -0.4637 | 0.10178 | 0.39644 | 0.115008 | -0.31516 |
| TREHALOSE | -0.48108 | 0.278462 | -0.2864 | 0.09526 | 0.398566 |
| PHOSPHORIC ACID | -0.48553 | 0.272272 | -0.1507 | -0.47086 | 0.101622 |
| GENTISIC ACID 5-O-GLUCOSIDE | -0.48862 | 0.404564 | 0.126389 | 0.044693 | 0.203452 |
| L-SORBOSE | -0.49143 | 0.127291 | -0.11497 | 0.182066 | 0.051185 |
| SUCROSE | -0.49427 | 0.274068 | -0.28472 | 0.084103 | 0.356827 |
| VITAMIN K1 | -0.49702 | 0.14157 | 0.093353 | -0.00152 | 0.221547 |
| (5S)-1,2,3,4,5,6,7,8,9,10-DECAHYDRO-5,9-METHANOBENZO[8]ANNULEN-11-ONE | -0.49986 | 0.15699 | 0.024254 | 0.015643 | 0.292796 |
| N-ACETYLALANINE | -0.50812 | 0.095741 | 0.037713 | 0.035854 | 0.212776 |
| FA 18:4+1O | -0.50814 | 0.059569 | 0.374439 | 0.064226 | -0.33756 |
| CIS-PARINARIC ACID METHYL ESTER | -0.50838 | 0.095948 | 0.422357 | 0.0018 | -0.35099 |
| 4-CYANO-D-PHENYLALANINE | -0.50863 | -0.00312 | 0.007872 | 0.024663 | 0.238807 |
| SEDOHEPTULOSAN | -0.51096 | 0.110782 | -0.24197 | 0.305366 | 0.074748 |
| (3E)-4-(2-(HYDROXYMETHYL)-6,6-DIMETHYL-4-OXOCYCLOHEX-2-EN-1-YL)BUT-3-EN-2-YL BETA-D-GLUCOPYRANOSIDE | -0.52008 | 0.235786 | -0.36983 | 0.336086 | 0.346481 |
| METHYL 4-HYDROXYCINNAMATE | -0.52626 | 0.028633 | -0.12968 | 0.126828 | 0.272605 |
| N-BENZYL-N,N-DIMETHYL-1-HEXADECANAMINIUM CATION | -0.53833 | 0.191864 | 0.066924 | -0.10324 | -0.06544 |
| BENZYLDIMETHYLSTEARYLAMMONIUM CATION | -0.53856 | 0.173265 | 0.05021 | -0.03636 | -0.02301 |
| BENZYLDIMETHYLTETRADECYLAMMONIUM CATION | -0.54494 | 0.157316 | 0.004297 | -0.01551 | 0.054017 |
| BETA-DAMASCONE | -0.556 | 0.216658 | -0.03987 | 0.215495 | 0.432895 |
| HEXYL 6-O-PENTOPYRANOSYLHEXOPYRANOSIDE | -0.55771 | 0.0005 | 0.283546 | 0.027215 | -0.33296 |
| RAFFINOSE | -0.57135 | 0.108529 | -0.14482 | -0.13203 | 0.147518 |
| MALTOSE | -0.59704 | 0.170678 | -0.27651 | 0.000378 | 0.38978 |
| ALPHA,BETA-TREHALOSE | -0.60061 | 0.155939 | -0.29138 | -0.09251 | 0.367132 |
| DL-2,4-DIAMINOBUTYRIC ACID | -0.61093 | -0.05168 | 0.081637 | 0.00359 | 0.150272 |
| 3-AMINO-N-[2-(4-MORPHOLINYL)ETHYL]BENZAMIDE | -0.64526 | 0.20858 | -0.07566 | 0.453776 | 0.025297 |
| N-ALPHA-ACETYL-L-ORNITHINE | -0.64599 | 0.074953 | 0.003535 | -0.0219 | 0.242925 |
| L-ALPHA-GLYCERYLPHOSPHORYLCHOLINE | -0.66657 | 0.249921 | 0.029885 | -0.21497 | 0.065353 |
| BETAINE | -0.6683 | 0.250915 | -0.01989 | -0.22721 | -0.0268 |
| 2-ACETAMIDO-2-DEOXY-3-O-(ALPHA-L-FUCOPYRANOSYL)-D-GLUCOPYRANOSE | -0.6741 | 0.101869 | -0.15477 | -0.11625 | 0.175799 |
| N,N-DIMETHYLBENZYLAMINE | -0.68045 | 0.116382 | -0.00972 | -0.20042 | 0.170597 |
| 2-PHENYLETHYL 3-O-(4-CARBOXY-3-HYDROXY-3-METHYLBUTANOYL)-BETA-D-GLUCOPYRANOSIDE (C20H28O10) | -0.72784 | -0.0206 | -0.28059 | 0.117591 | -0.00188 |
| L-CARNITINE | -0.73057 | 0.257113 | -0.02097 | -0.22996 | -0.01076 |
| N-ACETYL-D-GALACTOSAMINITOL | -0.76774 | 0.197352 | -0.07893 | 0.019453 | 0.184028 |
